# Caspase-11–mediated hyperinflammation impairs CD8^+^ T cell immunity and viral clearance in severe SARS-CoV-2 infection

**DOI:** 10.1172/jci.insight.199896

**Published:** 2026-09-22

**Authors:** Mostafa M. Eltobgy, Mohamed M. Shamseldin, Owen D. Whitham, Heba M. Amer, Jeffrey R. Atkinson, Asmaa Badr, Jesse M. Hall, Gauruv Gupta, Yara Y. Hassan, Rabab El-Mergawy, Richard Perez, Sarah E. Faber, Maciej Pietrzak, Amy Webb, Xiaoli Zhang, Adam D. Kenney, Destiny Bissell, Jihad I. Omran, Shady Estfanous, Kylene P. Daily, Amir Yousif, Marisa R. Joldrichsen, Andrew McNamara, Mahesh KC, Mark E. Peeples, Emily A. Hemann, Hazem E. Ghoneim, Shahid M. Nimjee, Estelle Cormet-Boyaka, Jianrong Li, Prosper N. Boyaka, Jacob S. Yount, Benjamin M. Segal, Purnima Dubey, Amal O. Amer

**Affiliations:** 1 Department of Microbial Infection and Immunity, College of Medicine, The Ohio State University, Columbus, Ohio, USA.; 2 Department of Microbiology and Immunology, Faculty of Pharmacy, Helwan University, Cairo, Egypt.; 3 Department of Neurology, College of Medicine and Wexner Medical Center,; 4 Neuroscience Research Institute, and; 5 Department of Biomedical Informatics, The Ohio State University, Columbus, Ohio, USA.; 6 College of Nursing, University of South Florida, Tampa, Florida, USA.; 7 Department of Veterinary Biosciences, College of Veterinary Medicine, The Ohio State University, Columbus, Ohio, USA.; 8 Abigail Wexner Research Institute at Nationwide Children’s Hospital, Columbus, Ohio, USA.; 9 Infectious Diseases Institute and; 10 Department of Neurological Surgery, College of Medicine and Wexner Medical Center, The Ohio State University, Columbus, Ohio, USA.

**Keywords:** Immunology, Infectious disease, Inflammation, Adaptive immunity, COVID-19, Innate immunity

## Abstract

Severe SARS-CoV-2 infection is characterized by lung hyperinflammation, impaired IFN responses, and defective T cell activation, yet the molecular drivers of these immune dysregulations remain incompletely understood. Caspase-11 (CASP11), a key effector of the noncanonical inflammasome, has been shown to mediate an innate hyperinflammatory response and cytokine release in a mild SARS-CoV-2 infection model. However, the role played by CASP11 in severe SARS-CoV-2 disease and how it affects adaptive immunity has not been identified. Here, we found that CASP11 exacerbates severe SARS-CoV-2 pathogenesis by amplifying early innate immune responses while concurrently impairing antiviral CD8^+^ T cell immunity. Using global KO mice, hematopoietic BM chimeras, and Cx3cr1-expressing mononuclear phagocyte system cell–specific CASP11 deletion models, we show that targeting CASP11 reduces lung inflammation, promotes early NK cell–mediated IFN-γ production, and enhances robust virus-specific effector CD8^+^ T cell responses. This was associated with enhanced viral clearance and improved survival, even under lethal infection conditions. Importantly, CASP11-KO mice also exhibited faster resolution of postviral inflammation. These findings position CASP11 as a promising immunomodulatory target for acute and delayed manifestations of severe SARS-CoV-2 infection.

## Abstract 2

Blocking the inflammatory protein caspase-11 restored virus-fighting T cells and improved viral clearance in mice, pointing toward a new target for severe COVID-19.

## Introduction

SARS-CoV-2, the etiologic agent of COVID-19, is an enveloped, positive-sense, single-stranded RNA virus belonging to the *Betacoronavirus* genus (1). Although most SARS-CoV-2 infections result in mild, self-limited upper respiratory symptoms, a substantial subset of individuals develop severe respiratory disease that can rapidly progress to acute respiratory distress syndrome (ARDS), respiratory failure, and multiorgan damage, leading to millions of deaths worldwide (2–4).

Severe SARS-CoV-2 disease is associated with an initial exaggerated innate immune response characterized by excessive release of proinflammatory cytokines such as IL-1β, IL-6, and IL-8, often referred to as a “cytokine storm,” accompanied by marked neutrophil infiltration into the lungs (5, 6). This inflammatory response mediates lung tissue damage and can result in respiratory failure and systemic inflammation (7). However, there are other cytokines that have been shown to be suppressed in the setting of severe SARS-CoV-2 infection. Specifically, type I IFN activity is diminished in patients critically ill with SARS-CoV-2, which is associated with persistent viremia and an exacerbated inflammatory response (8). Additionally, IFN-γ responses are compromised in these patients (9, 10). This inadequate IFN response, along with the plethora of proinflammatory cytokines, interferes with initiating an adequate and effective adaptive immune response in patients with severe SARS-CoV-2 disease (2, 11).

Patients with severe SARS-CoV-2 infection exhibit suboptimal T cell responses, marked by quantitative and qualitative alterations in both CD4^+^ and CD8^+^ T cell compartments (12–19). Several clinical studies have reported marked reductions in overall lymphocyte counts (lymphopenia), which correlate with disease severity and poor clinical outcomes (20, 21). Additionally, severe SARS-CoV-2 infection is associated with notable phenotypic alterations within the T cell pool, including increased T cell exhaustion, impaired activation and cytokine production, and disrupted T cell subset distribution (17, 18). However, the exact mechanisms driving T cell response impairment remain unclear. These alterations could result from direct viral invasion, prolonged antigen exposure, or indirect virus-induced excessive innate immune activation. Limited mechanistic and longitudinal studies have hindered a comprehensive understanding of these processes.

The inflammasome is a critical component of the innate immune system that plays a major role in SARS-CoV-2 infection (22–27). Among the best-characterized inflammasomes is the NLRP3 inflammasome, which is activated by a range of viral and host-derived danger signals during SARS-CoV-2 infection. Upon activation, NLRP3 recruits the adaptor protein ASC, leading to the activation of caspase-1, a canonical inflammasome effector protease. Caspase-1 cleaves proinflammatory cytokines IL-1β and IL-18 into their active forms and induces pyroptosis, a form of inflammatory cell death, thereby shaping the early inflammatory phase of SARS-CoV-2 infection (26, 28, 29).

Caspase-11 (CASP11) and its human homologs caspase-4/5 are critical members of the noncanonical inflammasome (30, 31). We previously showed that the lack of CASP11 decreases the innate inflammatory responses during moderate SARS-CoV-2 infection without affecting viral replication in the lung (32). In contrast, the lack of other inflammasome components such as NLRP3 or caspase-1 attenuates inflammation but promotes viral replication (27). CASP11 activation substantially amplifies inflammation by inducing cytokine production and pyroptotic cell death, suggesting its potential role in dysregulating subsequent adaptive immune responses (32–34). We previously demonstrated that CASP11 mediates cytokine release, neutrophil infiltration, and neutrophil extracellular trap (NET) formation after moderate SARS-CoV-2 infection (32). Additionally, we observed elevated CASP11 expression in lung tissue from humans with severe SARS-CoV-2 infection (32). However, the role of CASP11 in severe SARS-CoV-2 infection has not been well investigated, particularly regarding adaptive immune responses. Here, we found that a CASP11-mediated innate inflammatory response compromises an effective adaptive T cell response, contributing to severe disease outcomes in SARS-CoV-2 infection.

We mechanistically defined that CASP11 mediates its effects on both innate and adaptive immune responses after SARS-CoV-2 infection through its role in monocyte-derived cells. CASP11 exacerbates lung inflammation, suppresses IFN-γ signaling, and hinders the development of robust virus-specific effector CD8^+^ T cell responses.

## Results

### CASP11 deficiency protects against severe SARS-CoV-2 infection, limits early hyperinflammation, and enhances IFN-γ response.

**Figure 1:**
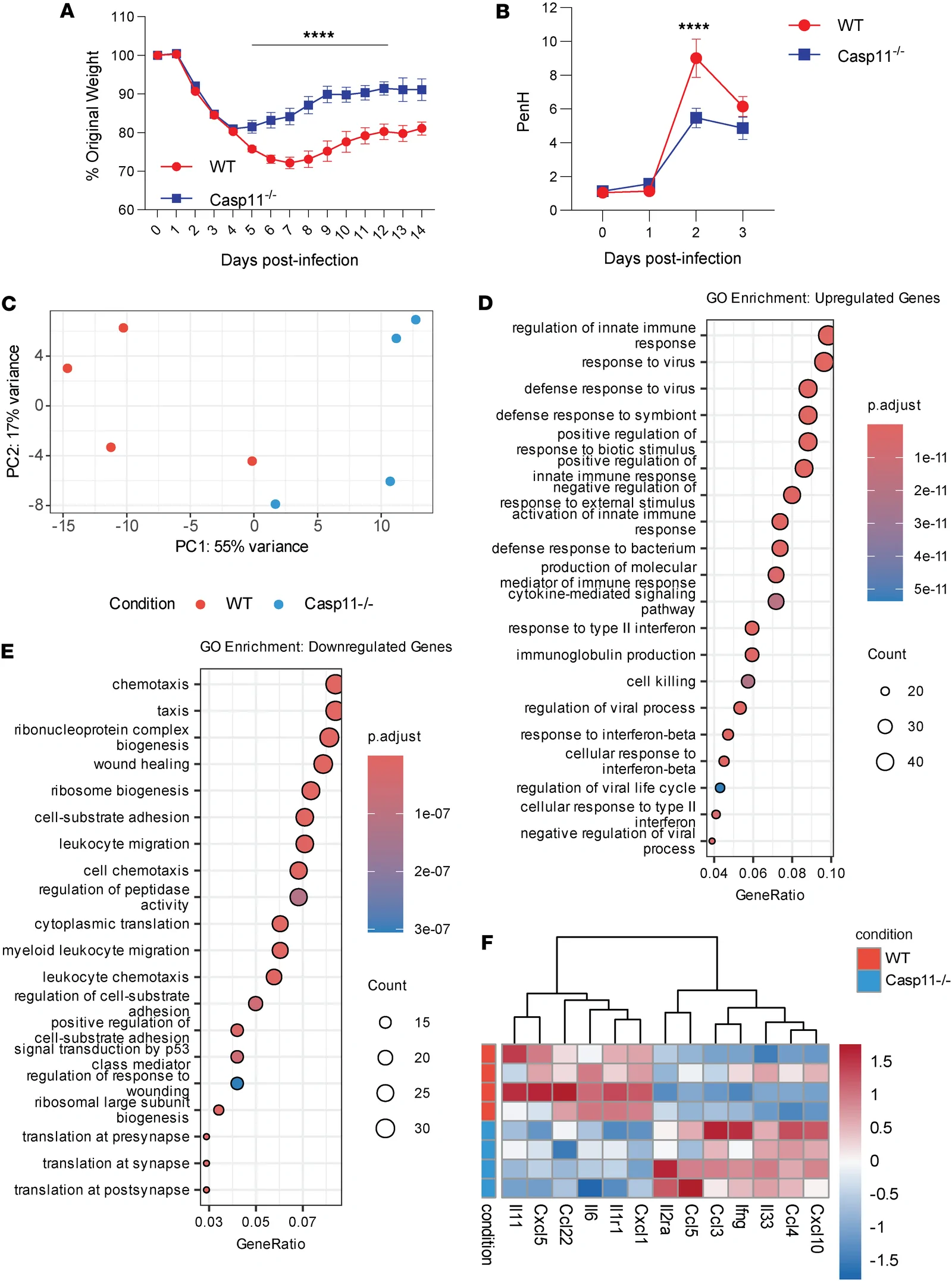
CASP11 deficiency protects against severe SARS-CoV-2 infection, limits early hyperinflammation, and enhances IFN-γ responses. (**A**) Body weight change in WT and Casp11^–/–^ mice after i.n. infection with 5 × 10^5^ TCID_50_ of MA10 SARS-CoV-2. WT mice exhibited more severe weight loss and slower recovery through day 14 compared with Casp11^–/–^ mice (*n* = 26/group). Statistical analysis: 1-way ANOVA with Šídák’s multiple-comparison test. (**B**) PenH, as a surrogate of airway resistance, measured by whole-body plethysmography at multiple time points after infection, showing significantly lower resistance in Casp11^–/–^ mice, indicative of improved lung function (*n* = 10 WT, *n* = 12 Casp11^–/–^). Statistical analysis: 2-way ANOVA with Šídák’s multiple-comparison test. (**C**) Principal component analysis (PCA) of variance-stabilized RNA-Seq data revealed distinct transcriptional profiles between WT and Casp11^–/–^ lungs at day 4 after infection (5 × 10^5^ TCID_50_ of MA10). PC1 accounted for 55% and PC2 for 17% of total variance. PERMANOVA analysis confirmed statistically significant separation between groups (*R*^2^ = 0.343, *F* = 3.135, *P* = 0.05, 999 permutations), indicating that genotype accounts for 34.3% of total transcriptional variance. Homogeneity of group dispersions was confirmed by betadisper analysis (*F* = 0.205, *P* = 0.554), validating the PERMANOVA result (*n* = 4/group). (**D**) GO enrichment analysis of genes with higher gene expression in Casp11^–/–^ lungs compared with WT reveals enrichment in IFN signaling pathways and antiviral immune pathways (*n* = 4/group). (**E**) GO enrichment analysis of genes with lower gene expression in Casp11^–/–^ lungs compared with WT highlights suppression of innate immune pathways, including myeloid cell chemotaxis and migration (*n* = 4/group). (**F**) Heatmap of differentially expressed cytokine and chemokine genes shows a shift in Casp11^–/–^ lungs from pro-myeloid inflammation (e.g., *Cxcl1*, *Il6*, *Ccl22*) to lymphocyte-promoting responses (e.g., *Ccl3*, *Ccl5*, *Ifng*) (*n* = 4/group). ****P < 0.0001.

We infected 12–16-week-old WT and CASP11 KO (Casp11^–/–^) mice with 5 × 10^5^ TCID_50_ of the MA10 mouse-adapted SARS-CoV-2 (35). WT mice developed severe disease, losing approximately 30% of their body weight by day 7 after infection (Figure 1A). In contrast, Casp11^–/–^ mice showed significantly milder disease, with recovery and notable weight regain starting at day 4 and continuing through day 14 after infection (Figure 1A). Daily body temperature monitoring revealed that Casp11^–/–^ mice exhibited a transient but significant decrease in body temperature at day 2 after infection compared with WT controls (Supplemental Figure 1A; supplemental material available online with this article; https://doi.org/10.1172/jci.insight.199896DS1), although no significant differences were observed between groups at later time points. We next evaluated pulmonary function using whole-body plethysmography, a noninvasive method that measures enhanced pause (PenH) as a surrogate indicator of airway resistance in conscious, unrestrained mice. Casp11^–/–^ mice exhibited significantly lower PenH compared with WT mice, indicating less respiratory distress and better lung function during infection (Figure 1B). This shows that in the absence of CASP11, mice are protected against clinically severe SARS-CoV-2 infection.

To further investigate the molecular mechanisms underlying the enhanced protection and recovery observed in Casp11^–/–^ mice, we performed bulk RNA-Seq of lung tissue harvested at day 4 after infection. Principal component analysis (PCA) revealed distinct transcriptional profiles between infected WT and Casp11^–/–^ lungs, highlighting a divergent host response (Figure 1C). PERMANOVA analysis confirmed statistically significant separation between groups (*R*^2^ = 0.343, *F* = 3.135, *P* = 0.05, 999 permutations), indicating that genotype accounts for 34.3% of total transcriptional variance. Homogeneity of group dispersions was confirmed by betadisper analysis (*F* = 0.205, *P* = 0.554), validating that the observed separation reflects true differences in group centroids rather than differences in within-group variance.

Gene Ontology (GO) enrichment analysis of genes with higher expression in the Casp11^–/–^ group showed a marked enrichment of antiviral immune pathways. Notably, both type I and type II IFN response pathways were significantly upregulated at the transcription level, suggesting a robust antiviral state in the absence of CASP11 (Figure 1D). In contrast, genes with lower expression in the Casp11^–/–^ lungs were predominantly related to myeloid cell chemotaxis and migration, suggesting a reduced innate inflammatory response in the absence of CASP11 (Figure 1E).

Further gene set enrichment analysis (GSEA) revealed significant enrichment of antiviral pathways in Casp11^–/–^ lungs, including the IFN-γ response and lymphocyte-mediated immunity (Supplemental Figure 1B). We then examined the expression of cytokines and chemokines in infected lungs. In Casp11^–/–^ mice, there was upregulation of IFN-γ and several chemokines known to promote effector T cell recruitment, including CCL3, CCL4, CCL5, and CXCL10 (36, 37) (Figure 1F and Supplemental Figure 2). In contrast, inflammatory mediators typically associated with neutrophil and monocyte recruitment such as CXCL1, CXCL5, CCL22, IL-6, IL-11, and IL1R1 were downregulated in Casp11^–/–^ lungs relative to WT (38, 39) (Figure 1F), suggesting a shift away from proinflammatory innate immune signaling toward a more lymphocyte-driven antiviral response (Figure 1F and Supplemental Figure 2).

**Figure 2:**
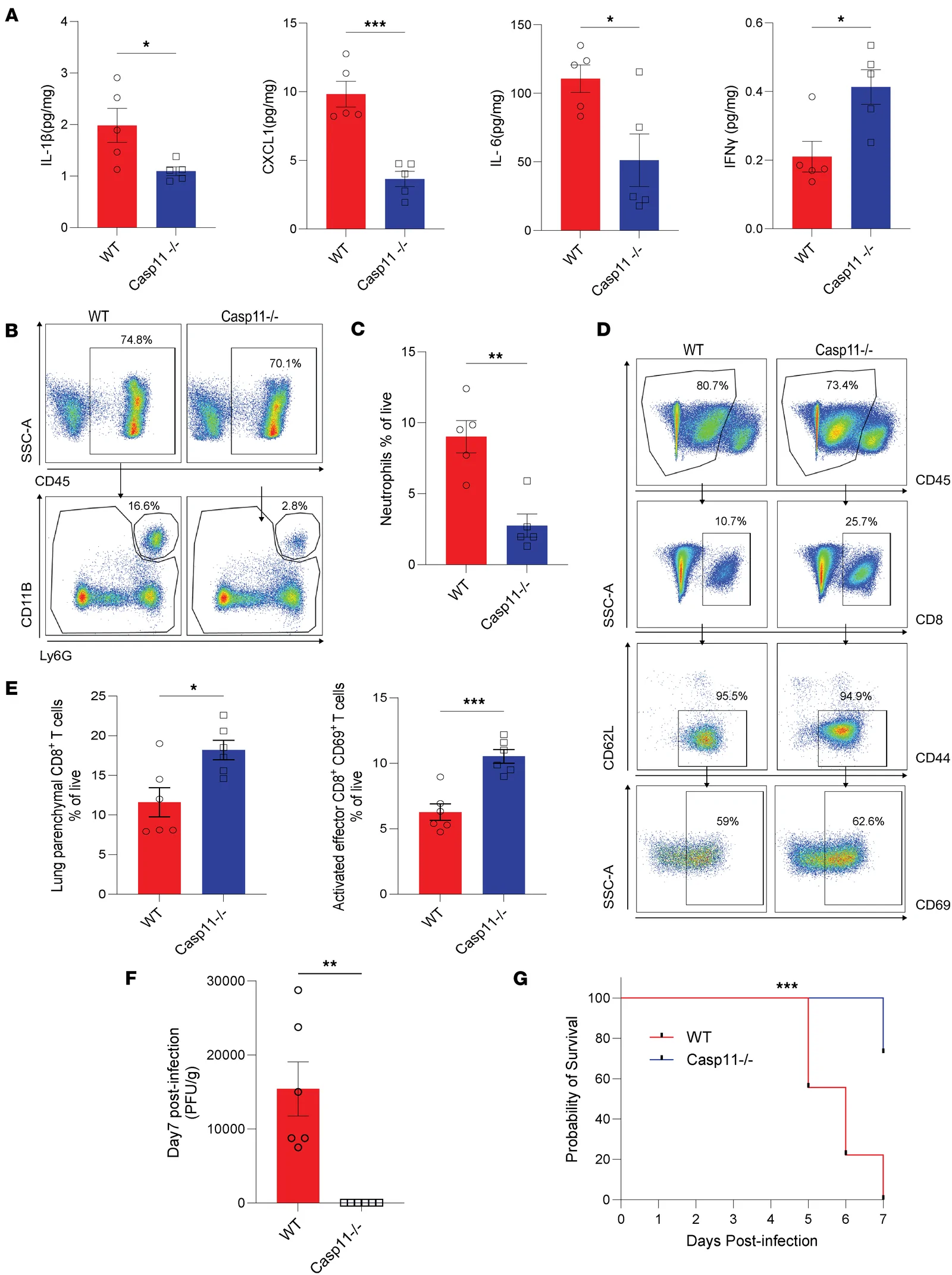
CASP11 deficiency limits early inflammation and neutrophil infiltration, enhances effector CD8^+^ T cell responses, promotes viral clearance, and protects against lethal SARS-CoV-2 infection. (**A**) Cytokine protein levels in lung homogenates at day 4 after infection (5 × 10^5^ TCID_50_ MA10), measured by ELISA and normalized to lung tissue weight (pg/mg tissue). Casp11^–/–^ lungs exhibited reduced levels of IL-1β, CXCL1, and IL-6, and elevated IFN-γ compared with WT (*n* = 5/group). Statistical significance was determined by unpaired Student’s *t* test. (**B** and **C**) Flow cytometry of lung tissue at day 4 after infection (5 × 10^5^ TCID_50_ MA10) shows significantly fewer neutrophils (CD45^hi^CD11b^hi^Ly6G^hi^) in Casp11^–/–^ mice compared with WT. Gating strategy (**B**) and quantification (**C**) (*n* = 5/group). Statistical significance was determined by unpaired Student’s *t* test. (**D** and **E**) At day 7 after infection, flow cytometry after intravascular anti-CD45 labeling revealed higher frequencies of lung parenchymal (CD45^lo^) CD8^+^ T cells and activated effector CD8^+^ T cells (CD62L^lo^CD44^hi^CD69^hi^) in Casp11^–/–^ lungs. Gating strategy (**D**) and quantification (**E**) (*n* = 6/group). Statistical significance was determined by unpaired Student’s *t* test. (**F**) Viral titers in lung homogenates assessed by plaque assay at day 7 after infection (5 × 10^5^ TCID_50_ MA10), expressed as PFU/g tissue. Casp11^–/–^ mice completely cleared the virus by day 7 in contrast to WT (*n* = 6/group). Statistical significance was determined by unpaired Student’s *t* test. (**G**) Kaplan-Meier survival analysis after high-dose SARS-CoV-2 challenge (1 × 10^6^ TCID_50_ MA10). All WT mice died by day 7; 72.7% of Casp11^–/–^ mice survived (*n* = 9 WT, *n* = 11 Casp11). Statistical significance was determined by log-rank (Mantel-Cox) test. *P < 0.05; **P < 0.01; ***P < 0.001.

We then examined cytokine protein levels in lung homogenates using ELISA at day 4 after infection, the same time point used for the RNA-Seq analysis. Compared with WT, Casp11^–/–^ lungs displayed markedly lower levels of IL-1β, CXCL1, and IL-6, and IFN-γ levels were significantly elevated (Figure 2A). These findings confirm the transcriptional signature observed by RNA-Seq and support a shift toward an enhanced IFN-γ response. We measured levels of type I IFNs (IFN-α and IFN-β) in lung homogenates at day 4 after infection using ELISA. We did not observe a significant difference in IFN-I protein levels between the WT and Casp11^–/–^ infected groups (Supplemental Figure 3A).

### CASP11 deficiency limits neutrophil infiltration, enhances effector CD8^+^ T cell responses, promotes viral clearance, and protects against lethal SARS-CoV-2 infection.

Given the distinct differences in immune responses between WT and Casp11^–/–^ mice, we next investigated the immune cell composition in the lungs after infection. Lungs were harvested on day 4 after infection and analyzed by flow cytometry. Consistent with the observed downregulation of neutrophil-attracting chemokines, Casp11^–/–^ lungs exhibited significantly reduced neutrophil infiltration compared with WT-infected lungs (Figure 2C, gating strategy shown in Figure 2B), supporting a reduced innate inflammatory response in the absence of CASP11.

Given the significantly elevated levels of IFN-γ protein in Casp11^–/–^ lungs at day 4 after infection, we sought to identify the primary cellular source of this cytokine during the early acute phase. Flow cytometric analysis with intracellular cytokine staining revealed that NK cells, rather than early infiltrating T cells, were the predominant producers of IFN-γ at this time point (Supplemental Figure 3, B and C).

Although both myeloid cells and NK cells were identified as baseline producers of IFN-γ at day 4 after infection, quantification revealed a compartment-specific enhancement in the Casp11^–/–^ mice. As shown in Supplemental Figure 3C, the frequency of IFN-γ^+^ NK cells was significantly increased in Casp11^–/–^ mice compared with WT controls. In contrast, IFN-γ production by myeloid cells and T cells remained unchanged between the genotypes. These data demonstrate that the CASP11-regulated elevation of early antiviral IFN-γ in Casp11^–/–^ mice is specifically attributable to NK cells.

We then performed flow cytometry on lung tissue harvested at day 7 after infection, a time point selected to allow for the development of an effector T cell response. To distinguish lung-resident (parenchymal) T cells from circulating peripheral T cells, mice were retro-orbitally injected with fluorescent anti-CD45 antibody 10 minutes before euthanization, labeling CD45^+^ cells in the intravascular space while leaving tissue-resident cells (lung parenchymal cells) unlabeled. Peripheral blood cells were more than 90% CD45^+^, demonstrating efficient labeling of circulating cells (data not shown). Flow cytometric analysis revealed that Casp11^–/–^ lungs contained significantly more lung parenchymal CD8^+^ T cells compared with WT controls (gating strategy in Figure 2, D and E). Further analysis of T cell activation status, based on surface marker expression, demonstrated an increased proportion of activated effector CD8^+^ T cells (CD62L^–^CD44^+^CD69^+^) in Casp11^–/–^ lungs relative to WT lungs (gating strategy in Figure 2, D and E). No significant differences were observed in CD4^+^ T cell populations between the 2 groups (Supplemental Figure 4A).

To test whether this enhanced T cell response translates into improved viral clearance, we performed plaque assays on lungs collected from WT and Casp11^–/–^ mice at days 4 and 7 after infection. Although viral loads were similar between groups at day 4 (Supplemental Figure 4B), by day 7, Casp11^–/–^ mice had completely cleared the virus, in contrast to WT mice, which still had detectable viral titers (Figure 2F). These results suggest that the robust activated effector CD8^+^ T cell response in Casp11^–/–^ mice is associated with enhanced viral clearance.

To gain a comprehensive understanding of the global adaptive immune response, we also evaluated the circulating intravascular CD8^+^ cell compartment, distinguished by in vivo intravascular CD45 labeling. Interestingly, in stark contrast to the enhanced effector response observed within the lung parenchyma, there were no significant differences between WT and Casp11^–/–^ mice in the frequencies of total circulating CD45^+^CD8^+^ T cells, CD69^+^ activated circulating CD8^+^ T cells, or IFN-γ^+^ circulating CD8^+^ T cells (Supplemental Figure 4, C–E).

We next asked whether CASP11 deficiency protects against lethal SARS-CoV-2 infection. WT and Casp11^–/–^ mice were infected with a higher dose of MA10 (1 × 10^6^ TCID_50_). All WT mice (*n* = 9) died from the infection by day 7, whereas 72.7% of the Casp11^–/–^ mice (8 of 11 mice) survived (Figure 2G). These data demonstrate that CASP11 deficiency confers protection from lethal SARS-CoV-2 challenge.

### CASP11 deficiency promotes functional, antigen-specific CD8^+^ T cell response independent of SARS-CoV-2 disease severity.

Next, we investigated whether the absence of CASP11 similarly promotes a robust effector CD8^+^ T cell response during moderate SARS-CoV-2 infection. To address this, we used a low-dose infection model in which WT and Casp11^–/–^ mice were infected with 1 × 10^5^ TCID_50_ of the mouse-adapted SARS-CoV-2 strain MA10. As previously reported, both WT and Casp11^–/–^ mice began regaining weight by day 4 and continued to recover through day 7, although WT mice exhibited a slower recovery trajectory (Supplemental Figure 5A) (32). Casp11^–/–^ mice also had reduced early inflammatory cytokines (IL-1β, CXCL1, and IL-6) and decreased neutrophil infiltration at days 2 and 4 after infection (32). Importantly, viral loads were comparable between strains, becoming nearly undetectable by day 4 and fully cleared by day 7 (32).

To further evaluate the adaptive immune response in this less-severe SARS-CoV-2 infection model, we performed flow cytometry on lung tissue at day 7 after infection, a time point when both groups had cleared the virus. As in previous experiments, mice received a retro-orbital injection of fluorescent anti-CD45 antibody prior to euthanasia to label intravascular immune cells, enabling us to distinguish infiltrating lung parenchymal from intravascular T cells.

**Figure 3:**
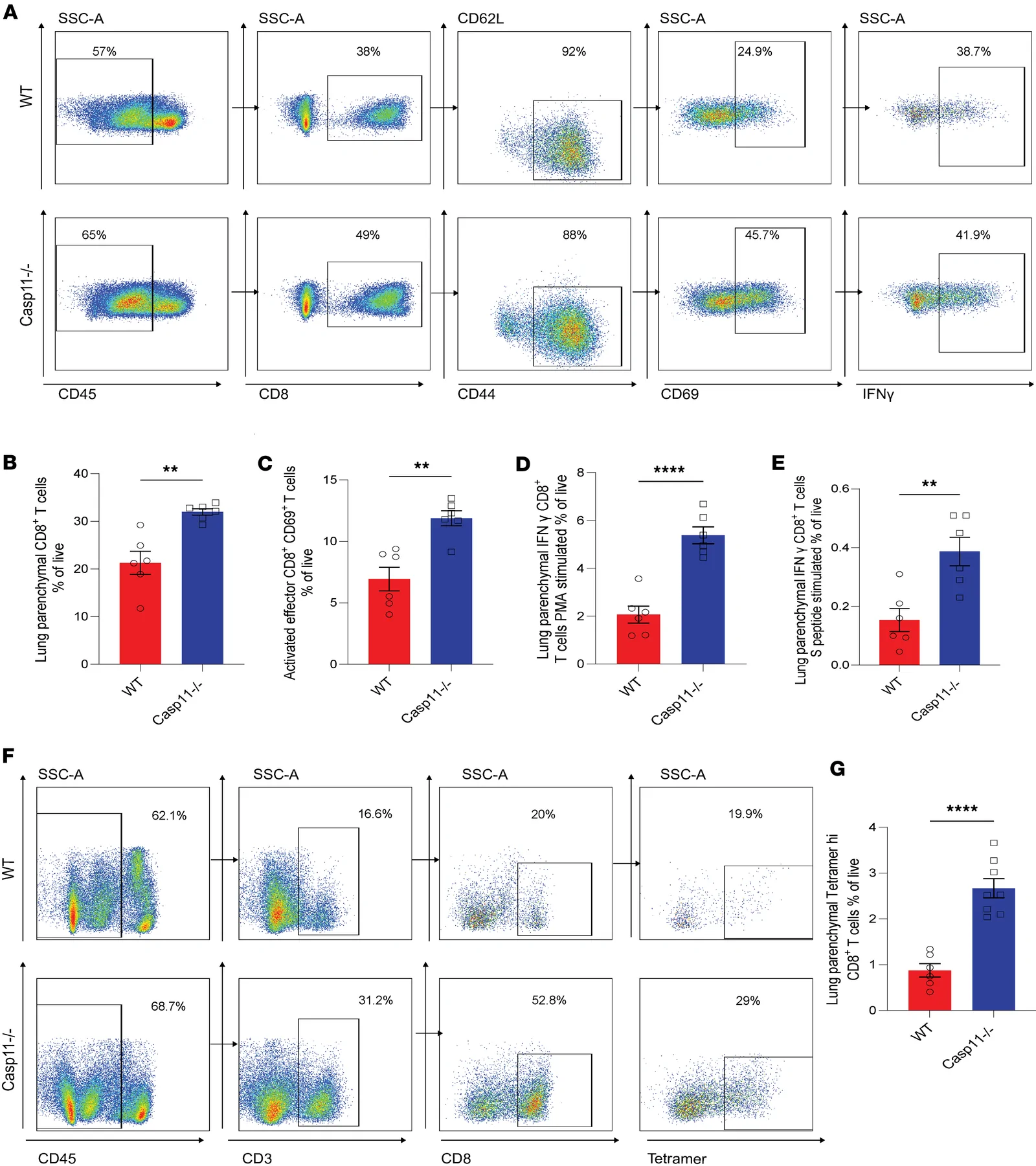
CASP11 deficiency promotes functional antigen-specific CD8^+^ T cell responses independent of SARS-CoV-2 disease severity. (**A**–**C**) Representative flow cytometry plots showing lung parenchymal CD8^+^ T cells (CD45^lo^CD8^+^) and activated effector CD8^+^ T cells (CD62L^lo^CD44^hi^CD69^hi^) at day 7 after infection in WT and Casp11^–/–^ mice following low-dose (1 × 10^5^ TCID_50_) MA10 SARS-CoV-2 infection. (**A**) Gating strategy. Quantification of lung parenchymal CD8^+^ T cells (**B**) and activated effector CD8^+^ T cells (**C**) as a percentage of live cells showing increased frequency of functional effector CD8^+^ T cells in Casp11^–/–^ lungs (*n* = 6/group). Statistical significance was determined by unpaired Student’s *t* test. (**D** and **E**) Flow cytometry analysis of intracellular IFN-γ production by CD8^+^ T cells after ex vivo stimulation with PMA/ionomycin (**D**) or SARS-CoV-2 spike peptide (**E**), showing increased frequency of functional IFN-γ–producing effector CD8^+^ T cells in Casp11^–/–^ lungs. Gating strategy shown in **A** (*n* = 6/group). Statistical significance was determined by unpaired Student’s *t* test. (**F** and **G**) Representative flow cytometry analysis of tetramer staining of lung CD8^+^ T cells using SARS-CoV-2–specific tetramer (H-2Db N219-227). (**F**) Gating strategy. (**G**) Quantification of tetramer^+^ CD8^+^ T cells among lung parenchymal T cells. Casp11^–/–^ mice exhibit a significantly greater population of antigen-specific CD8^+^ T cells (*n* = 6 WT, *n* = 8 Casp11^–/–^). Statistical significance was determined by unpaired Student’s *t* test. **P < 0.01; ****P < 0.0001.

We found that Casp11^–/–^ mice harbored a higher number of lung parenchymal CD8^+^ T cells compared with WT cells (Figure 3B, gating strategy shown in Figure 3A), including a significantly increased population of activated effector CD8^+^ T cells (CD62L^lo^CD44^hi^CD69^hi^) (Figure 3C, gating strategy shown in Figure 3A). These findings confirm that the enhanced CD8^+^ T cell response observed in Casp11^–/–^ mice is not merely a consequence of higher viral burden in WT mice. Instead, it is intrinsically driven by the absence of CASP11.

Because CASP11 is a central effector in the inflammasome pathway, we also investigated whether its absence affects the T cell phenotype in response to SARS-CoV-2 infection (40, 41). To explore this question, we isolated lungs from WT and Casp11^–/–^ mice at day 7 after infection and stimulated single-cell suspensions ex vivo with PMA/ionomycin or an overlapping peptide pool derived from SARS-CoV-2 spike protein. After stimulation, cells were stained for T cell activation markers and intracellular IFN-γ, since IFN-γ–producing CD8^+^ T cells are generally critical for respiratory virus clearance and infection control. Casp11^–/–^ mice showed increased numbers of IFN-γ–producing effector CD8^+^ T cells in response to both stimuli (Figure 3, D and E; gating strategy shown in Figure 3A), suggesting that Casp11 deficiency promotes expansion or maintenance of an antigen-specific CD8^+^ T cell population with heightened effector function.

Furthermore, to evaluate the antigen specificity of the CD8^+^ T cell response, we performed SARS-CoV-2 tetramer staining, which allows direct detection of CD8^+^ T cells specific for defined viral epitopes presented by MHC class I (42). This approach is especially useful for distinguishing true antigen-driven responses from bystander T cell activation, an important distinction in the context of viral infections like SARS-CoV-2. Casp11^–/–^ mice exhibited an increased number of lung parenchymal CD8^+^ T cells positive for tetramer (H-2D^b^ N_219-227_) (Figure 3G, gating strategy shown in Figure 3F), indicating that the enhanced CD8^+^ T cell response detected in the absence of CASP11 is composed of virus antigen-specific effector cells. Overall, these findings demonstrate that CASP11 deficiency enhances the quantity and functional quality of virus-specific effector CD8^+^ T cells in the lungs after SARS-CoV-2 infection.

### CASP11 mediates persistent inflammatory and T cell responses after SARS-CoV-2 infection, indicating ongoing inflammation and immune activation despite viral clearance.

Persistent inflammation and sustained T cell activation have been implicated in postviral sequelae, including tissue remodeling; immune dysregulation; and long-term complications such as fibrosis, impaired lung function, and long COVID (43–48). Therefore, we investigated whether CASP11 influences the persistence of inflammatory responses and immune cell activation after viral clearance and whether it contributes to the prolonged immune activity often observed beyond the virus replication phase in SARS-CoV-2 infection. To address this question, we infected WT and Casp11^–/–^ mice with MA10 (5 × 10^5^ TCID_50_). Mice were then euthanized at day 14, and lung tissue was collected for cytokine profiling and flow cytometric analysis. On day 14 after infection, WT lungs exhibited persistently elevated levels of inflammatory cytokines (Supplemental Figure 5B) and a higher number of CD8^+^ and CD4^+^ T cells than the lungs of Casp11^–/–^ mice (Supplemental Figure 5, C and D). In contrast, Casp11^–/–^ lungs demonstrated a more resolved immune state, characterized by lower cytokine levels and reduced T cell numbers.

To determine whether this sustained immunopathology correlates with chronic inflammasome activation, we analyzed *Casp11* mRNA expression at a late time point. Strikingly, qPCR analysis revealed a sustained upregulation of *Casp11* mRNA in the lung tissues of infected WT mice at 30 days after infection (Supplemental Figure 5E). Together, these findings indicate that *Casp11* expression persists long after viral clearance, suggesting a potential ongoing role in chronic immune dysregulation.

### CASP11 deficiency in hematopoietic immune cells enhances survival, reduces inflammation, and promotes T cell response after SARS-CoV-2 infection.

To further elucidate the mechanism by which CASP11 deficiency enhances the adaptive immune response and protects against severe disease, we generated hematopoietic BM chimeric mice (Supplemental Figure 6A). In this model, lethally irradiated WT recipient mice were reconstituted with BM from either WT or Casp11^–/–^ donors, generating 2 groups: WT → WT chimeras (WT chimera) and Casp11^–/–^ → WT chimeras (hereafter referred to as Casp11^–/–^ chimeras). This strategy enabled us to isolate the role of CASP11 within radiosensitive hematopoietic cells, such as monocytes, DCs, and lymphocytes, while preserving CASP11 expression in radioresistant nonhematopoietic compartments, including lung epithelial and endothelial cells. We assessed the contribution of hematopoietic CASP11 to SARS-CoV-2–induced immunopathology, T cell responses, and viral clearance. Importantly, interstitial macrophages and monocyte-derived macrophages in the lung are donor-derived, whereas alveolar macrophages are largely host-derived and remain WT due to their radioresistant, self-renewing nature (49). This setup enables a compartment-specific interpretation of CASP11 function in the lung immune microenvironment.

**Figure 4:**
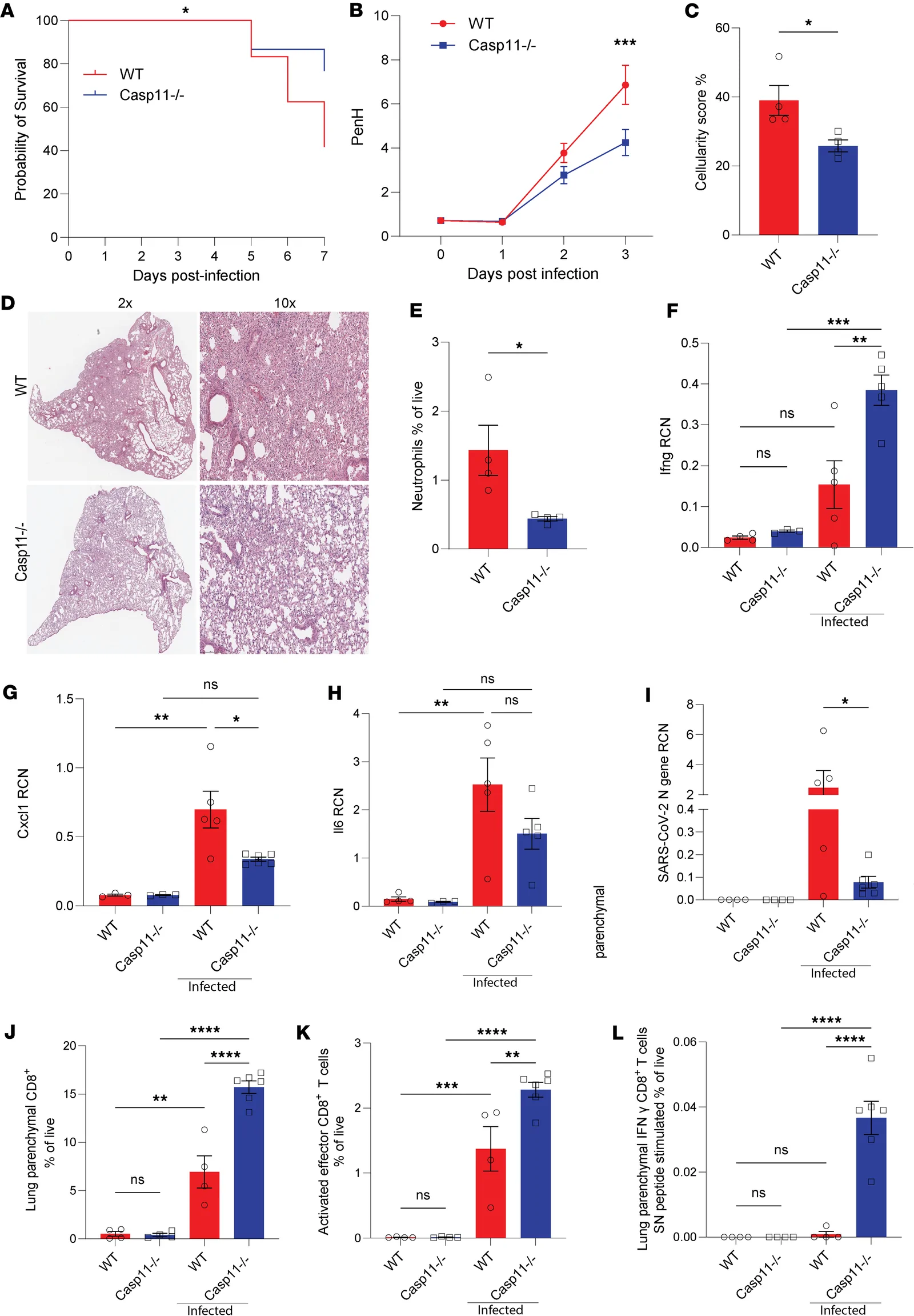
CASP11 deficiency in hematopoietic immune cells enhances survival, reduces inflammation, and promotes T cell response after SARS-CoV-2 infection. (**A**) Kaplan-Meier survival analysis of WT chimeras (WT → WT) and Casp11^–/–^ chimeras (Casp11^–/–^ → WT) after i.n. infection with 1 × 10^5^ TCID_50_ of MA10 SARS-CoV-2. Casp11^–/–^ chimeras (Casp11^–/–^ → WT) after i.n. infection with 1 × 10^5^ TCID50 of MA10 SARS-CoV-2(*n* = 24 WT chimeras, *n* = 30 Casp11^–/–^ chimeras). (**B**) PenH, a surrogate of airway resistance, measured by whole-body plethysmography at day 3 after infection (*n* = 18/group). (**C** and **D**) Cellularity scores (**C**) and representative H&E-stained lung sections (original magnification, ×2 and ×10) (**D**) at day 4 after infection (*n* = 4/group). (**E**) Flow cytometric quantification of neutrophils (LY6G^+^) in lung tissue at day 4 after infection (*n* = 4/group). (**F**–**H**) RT-qPCR analysis of inflammatory mediators in lung tissue at day 4 after infection Ifng (**F**) Cxcl1 (**G**) Il6 (**H**) in Casp11^–/–^ chimeras compared with WT chimeras (*n* = 5 infected WT chimeras, *n* = 6 infected Casp11^–/–^ chimeras, *n* = 3 noninfected WT chimeras, *n* = 3 noninfected Casp11^–/–^ chimeras). (**I**) SARS-CoV-2 N gene RNA copy number (RCN) in lung homogenates at day 7 after infection, quantified by RT-qPCR (*n* = 5 infected WT chimeras, *n* = 6 infected Casp11^–/–^ chimeras, *n* = 4 noninfected WT chimeras, *n* = 4 noninfected Casp11^–/–^ chimeras). (**J** and **K**) Flow cytometric quantification with intravascular CD45 labeling of lung parenchymal CD8^+^ T cells (**J**) and activated effector CD8^+^ T cells (CD62L^lo^CD44^hi^CD69^hi^) (**K**) at day 7 after infection (*n* = 4 infected WT chimeras, *n* = 6 infected Casp11^–/–^ chimeras, *n* = 4 noninfected WT chimeras, *n* = 4 noninfected Casp11^–/–^ chimeras). (**L**) Frequency of IFN-γ^+^ CD8^+^ T cells among lung parenchymal CD8^+^ T cells after ex vivo stimulation with SARS-CoV-2 SN peptide mixture at day 7 after infection. (*n* = 4 infected WT chimeras, *n* = 6 infected Casp11^–/–^ chimeras, *n* = 4 noninfected WT chimeras, *n* = 4 noninfected Casp11^–/–^ chimeras). Statistical significance determined by (**A**) log-rank (Mantel-Cox) test, (**B**) 2-way ANOVA with Šídák’s multiple-comparison test, (**C** and **E**) unpaired Student’s t test, and (**F**–**H**, **J**, **K**, and **L**) 1-way ANOVA with Tukey’s multiple-comparison test. Noninfected controls were age-matched, naive chimeric mice that underwent identical irradiation and BM reconstitution. Data shown as mean ± SEM. *P < 0.05; **P < 0.01; ***P < 0.001; ****P < 0.0001.

After a full reconstitution period, chimeric mice were infected with 1 × 10^5^ TCID_50_ of MA10. This dose, in the context of relatively aged chimeras (approximately 6 months old at infection), consistently induced severe disease. Only 41.7% (10 of 24) of WT chimeras survived by 7 days after infection, compared with 76.7% (23 of 30) of the Casp11^–/–^ chimeras (Figure 4A). Respiratory functions in infected mice were assessed by plethysmography, which revealed that Casp11^–/–^ infected chimera mice had decreased PenH values indicative of decreased airway resistance compared with infected WT chimeras (Figure 4B). This indicates that the deficiency of CASP11 in hematopoietic cells significantly alters lung functions and clinical severity in SARS-CoV-2 infection. Consistent with these findings, H&E staining of lung sections demonstrated that WT chimeras had significantly more lung consolidation, with increased edema, inflammation, and cellular infiltrates at day 4 after infection compared with Casp11^–/–^ chimeras (Figure 4. C and D). Flow cytometric profiling of lung myeloid cells at day 4 after infection showed fewer infiltrating neutrophils in Casp11^–/–^ chimeras compared with WT (Figure 4E).

Cytokine mRNA analysis by qPCR at day 4 after infection revealed *Ifng* was significantly upregulated while *Il6* and *Cxcl1* were downregulated in Casp11^–/–^ chimera mice (Figure 4, F–H), consistent with earlier observations in global KO. To investigate broader transcriptional changes, we performed RNA-Seq on lung tissue at day 4 after infection. GSEA revealed enrichment of gene sets related to T cell activation and antigen presentation pathways in Casp11^–/–^ chimeras (Supplemental Figure 6B).

By day 7 after infection, Casp11^–/–^ chimeras exhibited significantly lower SARS-CoV-2 N gene RNA copy numbers in the lungs compared with WT chimeras, indicating enhanced viral clearance (Figure 4I). To assess T cell response, we performed in vivo labeling of intravascular immune cells via retro-orbital injection of fluorescent anti-CD45 antibody, as previously described. This allowed us to selectively quantify lung parenchymal CD8^+^ T cells. Flow cytometric analysis revealed a higher number of parenchymal CD8^+^ T cells in Casp11^–/–^ chimeras relative to WT chimera controls (Figure 4J), along with a greater frequency of activated effector CD8^+^ T cells characterized by a CD62L^lo^CD44^hi^CD69^hi^ phenotype (Figure 4K).

To further evaluate CD8^+^ T cell effector function, we stimulated lung-derived CD8^+^ T cells ex vivo with a SARS-CoV-2 spike and nucleocapsid (SN) peptide mixture. Casp11^–/–^ chimeras demonstrated a higher frequency of IFN-γ–producing CD8^+^ T cells in response to ex vivo SN peptide stimulation, indicating preservation of effector function and enhanced antiviral potential (gating strategy shown in Supplemental Figure 7 and Figure 4L).

### Mononuclear phagocyte system cell–specific CASP11 deletion enhances antiviral CD8^+^ T cell responses, accelerates recovery, and improves viral clearance during SARS-CoV-2 infection.

The enhanced adaptive immune response observed in Casp11^–/–^ chimeras, along with reduced disease severity and improved viral clearance, suggests that CASP11 exerts its effects through hematopoietic immune cells. However, it remains unclear whether this regulation is primarily mediated by myeloid cells via modulation of the innate immune response, or whether CASP11 also plays a direct, intrinsic role in T cells.

To address this question, we generated mice with CASP11 deficiency specifically in Cx3cr1-expressing mononuclear phagocyte system (MPS) cells. We utilized Cx3cr1-CreERT2 mice, in which Cre-ERT2 and EYFP are knocked into the Cx3cr1 locus, to generate CASP11-deficient mice in Cx3cr1-expressing lineages. The main cell types targeted by this system include patrolling Ly6C-LOW monocytes, interstitial macrophages, monocyte-derived macrophages, and a subset of DCs, all of which express CX3CR1 at moderate-to-high levels (50). Classical Ly6C^hi^ inflammatory monocytes, which express lower levels of CX3CR1, are less efficiently targeted by this system. Alveolar macrophages are minimally affected due to their low CX3CR1 expression (50, 51). Neutrophils and lymphocytes do not express CX3CR1 at high levels and are therefore not targeted (50, 52).

Importantly, both control and experimental mice are homozygous for the Cx3cr1-CreERT2 knockin allele, rendering both groups CX3CR1-deficient (Supplemental Figure 8A). This shared genetic background ensures that any observed differences between groups are attributable specifically to CASP11 deletion rather than CX3CR1 deficiency. For simplicity, we refer to Cx3cr1-CreERT2 Casp11^WT/WT^ mice as Cx3cr1-Cre control and Cx3cr1-CreERT2 Casp11^fl/fl^ mice as Cx3cr1-MPS-Casp11^–/–^.

To validate the specificity and efficiency of our inducible KO model, we utilized the YFP fluorescent reporter to track Cre recombinase activity in Cx3cr1-expressing cells. Flow cytometric analysis of noninfected lungs from Cx3cr1-Cre control and Cx3cr1-MPS-Casp11^–/–^ mice was performed using a gating strategy that sequentially excluded dead cells and LY6G^+^ neutrophils, followed by identification of CD11B^+^F480^+^ macrophages and CD4^+^ and CD8^+^ T cells (Supplemental Figure 8B).

This analysis confirmed robust and selective YFP reporter expression specifically within the CD11B^+^F480^+^ lung macrophage compartment, which includes interstitial macrophages and monocyte-derived macrophages (95% YFP^+^). Conversely, LY6G^+^ neutrophils (4.5% YFP^+^), CD4^+^ T cells (0.5% YFP^+^), and CD8^+^ T cells (3.3% YFP^+^) remained largely YFP^–^, confirming precise cellular targeting within the mononuclear phagocyte compartment and a lack of appreciable off-target recombination in innate or adaptive immune cells (Supplemental Figure 8C).

To functionally verify targeted CASP11 deletion during acute infection, YFP^+^ cells were FACS-sorted from lung tissue at day 3 after SARS-CoV-2 infection. qPCR confirmed an approximately 75% reduction in Casp11 mRNA expression specifically within the sorted YFP^+^ macrophage compartment of Cx3cr1-MPS-Casp11^–/–^ mice relative to Cx3cr1-Cre matched controls (Supplemental Figure 8D). Together, these data demonstrate specific and efficient CASP11 deletion within the Cx3cr1-expressing lung macrophage compartment, with no substantial targeting of neutrophils or lymphocytes.

**Figure 5:**
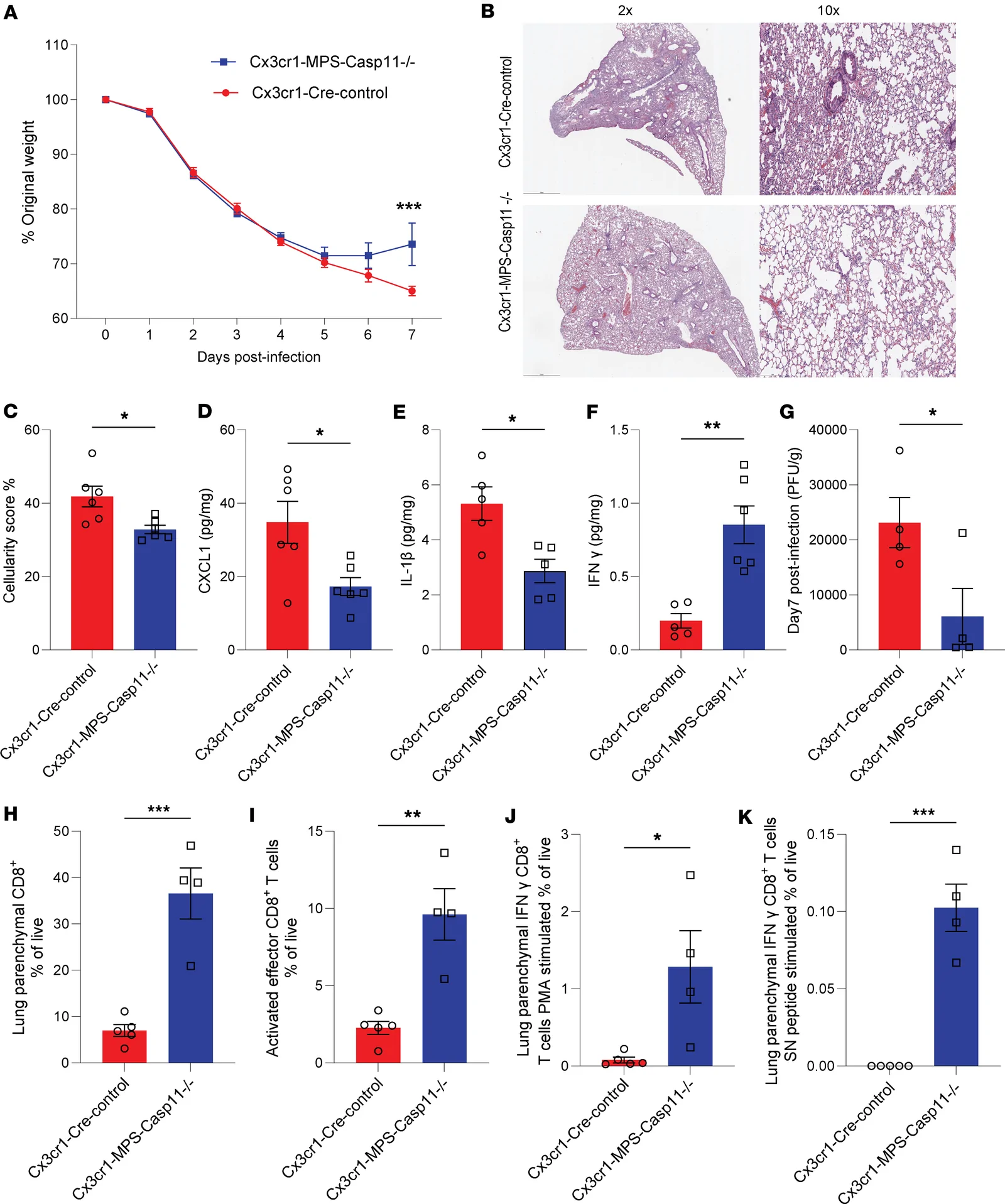
Cx3cr1-expressing MPS cell-specific CASP11 deletion enhances antiviral CD8^+^ T cell responses, accelerates recovery, and improves viral clearance during SARS-CoV-2 infection. (**A**) Body weight kinetics in Cx3cr1-Cre control and Cx3cr1-MPS-Casp11^–/–^ mice after i.n. infection with 5 × 10^5^ TCID_50_ of MA10 SARS-CoV-2. Cx3cr1-MPS-Casp11^–/–^ mice showed significantly improved recovery compared with Cx3cr1-Cre controls (*n* = 6/ Cx3cr1-Cre controls, *n* = 11/ Cx3cr1-MPS-Casp11^–/–^). (**B**) Representative H&E-stained lung sections at day 4 after infection showing reduced inflammatory infiltrates and edema in Cx3cr1-MPS-Casp11^–/–^ mice versus Cx3cr1-Cre controls. Original magnification, ×2 and ×10. (**C**) Histopathological cellularity scoring of H&E-stained lung sections confirms significantly reduced pulmonary inflammation in Cx3cr1-MPS-Casp11^–/–^ mice compared with Cx3cr1-Cre controls at day 4 after infection (*n* = 6/group). (**D**–**F**) Cytokine protein levels measured by ELISA in lung homogenates at day 4 after infection. Cx3cr1-MPS-Casp11^–/–^ mice demonstrate significantly reduced CXCL1 (**D**, pg/mg tissue) and IL-1β (**E**, pg/mg tissue), and elevated IFN-γ (**F**, pg/mg tissue) compared with Cx3cr1-Cre controls (*n* = 6). (**G**) Viral titers in lung homogenates at day 7 after infection quantified by plaque assay on Vero E6-TMPRSS2 cells reveal significantly lower viral loads in Cx3cr1-MPS-Casp11^–/–^ mice compared with Cx3cr1-Cre controls (PFU/g) (*n* = 4). (**H**) Quantification of lung parenchymal CD8^+^ T cells (CD45^–^CD8^+^) by flow cytometry with intravascular CD45 labeling at day 7 after infection. Cx3cr1-MPS-Casp11^–/–^ mice harbor significantly greater numbers of lung parenchymal CD8^+^ T cells compared with Cx3cr1-Cre controls (*n* = 5/Cx3cr1-Cre controls, *n* = 4/Cx3cr1-MPS-Casp11^–/–^). (**I**) Frequency of activated effector CD8^+^ T cells (CD62L^lo^ CD44^hi^ CD69^hi^) among lung parenchymal CD8^+^ T cells at day 7 after infection. Cx3cr1-MPS-Casp11^–/–^ mice show a significantly higher proportion of activated effector CD8^+^ T cells than Cx3cr1-Cre controls (*n* = 5/Cx3cr1-Cre controls, *n* = 4/Cx3cr1-MPS-Casp11^–/–^). (**J** and **K**) Frequency of IFN-γ^+^ CD8^+^ T cells among lung parenchymal CD8^+^ T cells after ex vivo stimulation with PMA/ionomycin (**J**) or SARS-CoV-2 SN peptide mixture (**K**) at day 7 after infection. Cx3cr1-MPS-Casp11^–/–^ mice demonstrate a significantly higher frequency of IFN-γ–producing CD8^+^ T cells than Cx3cr1-Cre controls under both stimulation conditions (*n* = 5/Cx3cr1-Cre controls, *n* = 4/Cx3cr1-MPS-Casp11^–/–^). Statistical significance determined by (**A**) 2-way ANOVA with Šídák’s multiple-comparison test and (**C**–**K**) unpaired Student’s *t* test. Data shown as mean ± SEM. *P < 0.05; **P < 0.01; ***P < 0.001; ****P < 0.0001.

Cx3cr1-MPS-Casp11^–/–^ and Cx3cr1-Cre control mice were infected with 5 × 10^5^ TCID_50_ of MA10 virus to establish a severe SARS-CoV-2 infection model. Cx3cr1-MPS-Casp11^–/–^ mice exhibited enhanced recovery, indicating milder disease severity (Figure 5A). Histological analysis (H&E staining) and cellularity scoring of lung sections at day 4 after infection revealed reduced inflammatory infiltrates and edema in Cx3cr1-MPS-Casp11^–/–^ mice compared with Cx3cr1-Cre controls (Figure 5, B and C).

We next investigated the cytokine release profile in lung tissue harvested at day 4 after infection. Cx3cr1-MPS-Casp11^–/–^ mice showed reduced release of inflammatory cytokines, including CXCL1 and IL-1β (Figure 5, D and E), while exhibiting elevated levels of IFN-γ (Figure 5F), consistent with observations in global Casp11^–/–^ KO and chimera mice. These findings confirm that modulation of the early innate immune response and the resulting lung protection are mainly mediated by CASP11 within MPS cells.

We then used a plaque assay to quantify the viral load in the lungs of infected mice at day 7 after infection. We found that Cx3cr1-MPS-Casp11^–/–^ mice had significantly lower viral loads compared with Cx3cr1-Cre control mice, indicating that the enhanced viral clearance observed in global Casp11^–/–^ KO mice and Casp11^–/–^ chimeras is due to the immunomodulatory effect of CASP11 in monocyte-derived cells (Figure 5G).

Next, we investigated changes in the CD8^+^ T cell response mediated by Casp11 deficiency in mononuclear phagocyte cells. Cx3cr1-MPS-Casp11^–/–^ and Cx3cr1-Cre control mice were infected with the same dose of MA10 (5 × 10^5^ TCID_50_) and euthanized at day 7 after infection, after retro-orbital injection of fluorescent anti-CD45 antibody, as described before. Single-cell suspensions were prepared from the lungs, stained for surface markers, and subsequently stimulated ex vivo with PMA/ionomycin and a mixture of SN peptides, followed by permeabilization and intracellular IFN-γ staining. Flow cytometric analysis revealed that Cx3cr1-MPS-Casp11^–/–^ mice had a higher number of lung parenchymal CD8^+^ T cells compared with Cx3cr1-Cre control mice (Figure 5H) (gating strategy shown in Supplemental Figure 9). Additionally, analysis of CD69^+^ activated effector CD8^+^ T cells showed significantly increased numbers in Cx3cr1-MPS-Casp11^–/–^ mice (Figure 5I).

Intracellular cytokine staining demonstrated a higher frequency of IFN-γ–producing CD8^+^ T cells in the lungs of Cx3cr1-MPS-Casp11^–/–^ mice compared with Cx3cr1-Cre control mice after stimulation with both PMA/ionomycin and SN peptide mixtures (Figure 5, J and K) (gating strategy shown in Supplemental Figure 9). Moreover, bulk RNA-Seq of lung tissue at day 4 after infection, coupled with GSEA, revealed enhanced antigen presentation and upregulation of lymphocyte activation and T cell activation pathways (Supplemental Figure 10). Collectively, these findings demonstrate that the upregulated IFN-γ response and the more robust adaptive CD8^+^ T cell response, leading to accelerated recovery and viral clearance, are mediated by the absence of CASP11 in myeloid cells, primarily lung interstitial macrophages and monocyte-derived macrophages.

## Discussion

The absence of effective therapies addressing both viral replication and immune dysregulation remains a substantial clinical gap in SARS-CoV-2 acute and postacute conditions. Although previous clinical reports have documented suboptimal and delayed adaptive T cell responses in patients with severe SARS-CoV-2 infection, the underlying mechanisms driving these impaired T cell responses remain unclear (12–19). Although much attention has been given to the hyperinflammatory responses driving severe SARS-CoV-2 infection, the mechanisms by which this dysregulation undermines antiviral T cell immunity remain poorly understood (5, 14, 53–59).

Our findings suggest that targeting CASP11 can effectively address this gap. CASP11 deficiency enhances early tissue-resident effector CD8^+^ T cell responses, without functional impairments in T cells, such as altered phenotype, diminished cytokine production, or compromised antigen specificity, leading to protection from severe SARS-CoV-2 infection and more efficient viral clearance. These results uncover an unexpected role for CASP11 in impairing adaptive immunity, extending its known function beyond innate immune activation and pyroptosis. CASP11 deficiency shifts lung immunity from innate hyperinflammation toward an enhanced, early, protective adaptive T cell response, leading to more efficient viral clearance. Specifically, Casp11^–/–^ mice exhibited a robust IFN-γ response with enhanced expression of T cell–recruiting chemokines (CCL3, CCL4, CCL5, and CXCL10), which facilitated CD8^+^ T cell infiltration and/or proliferation in infected lungs.

Mounting a robust IFN response early during SARS-CoV-2 infection is crucial for establishing effective antiviral immunity, as impaired IFN signaling has been detected in patients with severe SARS-CoV-2 infection (60–62). Moreover, several studies have reported diminished and delayed IFN-γ responses in severe SARS-CoV-2 infection, correlated strongly with impaired T cell functionality, increased disease severity, and viral persistence (63–66). Our findings align with and expand upon these reports, demonstrating that targeting CASP11 is a promising strategy to enhance IFN-γ response, which rapidly facilitates a shift toward an effective antiviral CD8^+^ T cell–mediated response. IFN-γ is critically involved in the upregulation of chemokines responsible for recruiting and activating T cells, like CXCL10, as well as directly skewing immune responses toward CD8^+^ cytotoxic T cells (67–69). Casp11^–/–^ mice exhibited a potent T cell–favoring chemokine signature, including upregulated expression of CCL3, CCL4, CCL5, and CXCL10 (36, 37). These chemokines are known to interact with chemokine receptors, primarily expressed on CD8^+^ T cells, thereby promoting efficient T cell recruitment, proliferation, and differentiation into tissue-resident memory cells. This provides a mechanistic link between innate immune response, enhanced IFN-γ signaling, chemokine-driven CD8^+^ T cell responses, and improved viral clearance mediated by the absence of CASP11.

The profound shift in the pulmonary chemokine landscape observed in the absence of CASP11 highlights important crosstalk between the MPS, innate lymphoid cells, and lung microenvironment. In severe SARS-CoV-2 infection, early viral sensing by WT MPS cells triggers robust CASP11 activation, driving a profound hyperinflammatory response characterized by the massive release of early mediators like IL-1β and CXCL1, which is further exacerbated by inflammatory cell death (pyroptosis), resulting in abundant neutrophil infiltration. We previously demonstrated that CASP11 is also required for NET formation during SARS-CoV-2 infection (32). This innate influx creates a highly suppressive environment, as persistently elevated neutrophils and excessive NETs have been shown to directly hinder T cell activation and proliferation (70).

Conversely, targeted deletion of CASP11 within the MPS compartment blunts this initial hyperinflammatory cascade, significantly reducing neutrophil infiltration through day 4 after infection. This allows the MPS cells to transition toward a protective antiviral signaling state. This altered microenvironment facilitates the early activation and/or recruitment of NK cells, which we identified as the CASP11-regulated cellular source of the significantly elevated IFN-γ observed in Casp11^–/–^ lungs at day 4 after infection. This robust, early wave of NK cell–derived IFN-γ acts via potent autocrine and paracrine signaling pathways, likely involving both immune cells and adjacent lung epithelial and endothelial cells, to drive the secondary transcription of T cell–recruiting chemokines, including CXCL10, CCL3, CCL4, and CCL5 (71–73). Thus, deletion of CASP11 in MPS cells orchestrates an effective innate-to-adaptive immune relay, shifting the microenvironment away from suppressive, neutrophil-driven hyperinflammation and toward an IFN-γ–driven chemokine gradient that actively recruits and supports antiviral CD8^+^ T cells.

Crucially, our analysis of the circulating T cell compartment provides important mechanistic insight into how CASP11 modulates adaptive immunity. We observed no significant differences in the frequencies, activation status, or IFN-γ production of circulating intravascular (CD45^+^) CD8^+^ T cells between WT and Casp11^–/–^ mice. This highly compartmentalized difference, a robust enhancement in the lung parenchyma but no change in the circulation, indicates that CASP11 deficiency does not cause a systemic, nonspecific alteration in T cell response. This strongly supports our conclusion that the impaired CD8^+^ T cell response in WT mice is driven strictly by the local, hyperinflammatory, and suppressive lung microenvironment orchestrated by CASP11, which compromises the survival and function of recruited effector T cells once they enter the primary site of infection.

Although Casp11^–/–^ mice exhibited an upregulated type I IFN signature in our RNA-Seq analysis, subsequent protein analysis revealed no significant difference in type I IFN (IFN-α/β) levels between infected WT and Casp11^–/–^ lungs at day 4 after infection. Although early type I IFN signaling may contribute to the initial containment of viral replication, it is unlikely to fully account for the improved viral clearance observed later in the disease course. Notably, both WT and Casp11^–/–^ mice exhibited comparable viral loads at day 4 after infection, yet by day 7, Casp11^–/–^ mice showed a marked reduction in lung viral titers. This timeline strongly correlates with the expansion and activation of virus-specific CD8^+^ T cells, suggesting that adaptive immunity, rather than innate viral control alone, is the primary driver of improved viral clearance in the absence of CASP11. Furthermore, multiple studies have demonstrated that T cells, rather than type I IFNs, are essential for SARS-CoV-2 clearance in mouse models (74–76). For instance, Rag1^–/–^ mice, which lack functional T and B cells, fail to control SARS-CoV-2 replication and exhibit persistent viral loads despite intact innate responses (74, 75). These findings align with our longitudinal data analysis, supporting the conclusion that CASP11 deficiency facilitates late-stage viral clearance primarily by enhancing CD8^+^ T cell–mediated responses.

CASP11 deficiency and the consequent early recruitment of activated effector CD8^+^ T cells effectively controlled severe infection and significantly improved viral clearance. Even under conditions of high viral load intended to induce lethal disease, CASP11 deficiency markedly promoted survival, highlighting the therapeutic potential of specifically targeting CASP11. This contrasts with the published results of targeting other inflammasome components, such as caspase-1 or NLRP3, which resulted in an antiinflammatory effect and a reduction of disease severity but failed to control viral replication (27). Current available therapeutic interventions for severe SARS-CoV-2 infection primarily depend on immunosuppressive agents, such as corticosteroids and IL-6 inhibitors, which mitigate hyperinflammatory responses without directly promoting antiviral immunity or enhancing viral clearance (77–79). Therefore, CASP11 targeting represents a disease-modifying therapeutic strategy rather than merely an immunosuppressive approach.

Although a robust T cell response is essential for effective viral clearance, accumulating evidence suggests that persistent T cell activation may contribute to SARS-CoV-2 postviral pathology. In mouse models, the T cell response to SARS-CoV-2 was associated with increased lung inflammation and injury, despite improved viral clearance (74). Recent clinical studies have reported that individuals with long-term respiratory complications or long COVID exhibit prolonged SARS-CoV-2–specific T cell activation even months after viral clearance, in contrast to those who recover fully. This sustained immune activation has been implicated in chronic inflammation, impaired lung function, and tissue remodeling (43–48). Infected Casp11^–/–^ mice displayed resolution of the inflammatory response at day 14 after infection with no detectable cytokine production and fewer inflammatory T cells. In contrast, WT mice exhibited persistent proinflammatory cytokine expression at day 14 after infection and sustained activation of CD4^+^ and CD8^+^ T cells despite complete viral clearance. Additionally, we observed persistent *Casp11* mRNA upregulation in infected lungs up to 30 days after infection, suggesting sustained inflammasome activation well beyond the point of viral clearance. These findings suggest that CASP11 not only amplifies early inflammation but also impairs immune resolution, thereby contributing to prolonged immune activation and potential long-term tissue damage.

To further dissect the compartment-specific role of CASP11, we employed a hematopoietic BM chimera model. By transplanting BM from Casp11^–/–^ or WT donors into lethally irradiated WT hosts, we created mice in which only hematopoietic-derived cells lacked CASP11, while radioresistant structural cells, including epithelial, endothelial, and alveolar macrophages, retained WT expression. In this setting, Casp11^–/–^ chimeras recapitulated the protective phenotype seen in global KO, with enhanced survival, improved lung function, reduced inflammation, and more robust adaptive immunity after SARS-CoV-2 infection. Although the reciprocal chimeric model (WT hematopoietic cells transferred into Casp11^–/–^ hosts) would further delineate the baseline contribution of tissue-resident cells, our current chimera data strongly suggest that CASP11 expression in the radioresistant structural and resident macrophage compartments is insufficient to drive severe immunopathology on its own. The indispensable driver of severe disease resides within the recruited hematopoietic compartment. Yet, it remained unclear how CASP11, a molecule traditionally associated with innate immunity, could influence adaptive T cell responses and viral clearance. Although prior studies have hinted at intrinsic roles for CASP11 in CD8^+^ T cell biology (80), we resolved this question by employing Cx3cr1-MPS-Casp11^–/–^ mice, which selectively delete Casp11 in Cx3cr1-expressing MPS cells, including patrolling monocytes, interstitial macrophages, monocyte-derived macrophages, and a subset of DCs. This allowed us to isolate the effect of CASP11 in Cx3cr1-expressing myeloid cells without affecting the expression of Casp11 in lymphoid or structural compartments. Strikingly, even with CASP11 deletion confined to Cx3cr1-expressing myeloid cells, we observed an upregulated IFN-γ response, pronounced enhancement of CD8^+^ T cell activation, increased lung parenchymal CD8^+^ T cells, and a greater frequency of IFN-γ–producing effector cells. These findings argue against a cell-intrinsic role for CASP11 within CD8^+^ T cells and instead suggest that its immunomodulatory effects in SARS-CoV-2 are mediated through shaping the innate immune microenvironment.

We provide the first evidence, to our knowledge, that targeting CASP11 in Cx3cr1-expressing myeloid cells modulates the innate immune environment in a way that protects against severe SARS-CoV-2 infection while also enhancing the development of a robust IFN-γ response and CD8^+^ adaptive immune response, ultimately leading to more efficient viral clearance. These findings suggest that CASP11 acts as an immunomodulatory molecule, capable of dampening harmful inflammation without broadly suppressing host immunity, distinguishing it from current immunosuppressive interventions used in severe SARS-CoV-2 infection.

## Methods

### Sex as a biological variable.

Our infection studies utilized both male and female animals, and we observed similar outcomes for both male and female mice. Pooled male and female data are shown throughout the manuscript.

### Biosafety.

Most experiments involving live SARS-CoV-2 were conducted in The Ohio State University biosafety level 3 (BSL-3) biocontainment facility. All procedures were reviewed and approved by The Ohio State University BSL-3 Operations/Advisory Group, the Institutional Biosafety Officer, and the Institutional Biosafety Committee. After the reclassification of SARS-CoV-2 to BSL-2, subsequent experiments were performed in a BSL-2 biocontainment facility in accordance with The Ohio State University biosafety guidelines.

### Viruses and titers.

Mouse-adapted SARS-CoV-2, variant strain MA10 (35), generated by the laboratory of Ralph Baric (University of North Carolina) was provided by BEI Resources (NR-55329). Viral stocks from BEI Resources were plaque purified on Vero E6 cells to identify plaques lacking mutations in the polybasic cleavage site of the spike protein via sequencing. Nonmutated clones were propagated on Vero E6 cells stably expressing TMPRSS2 (provided by Shan-Lu Liu, The Ohio State University). Virus aliquots were flash-frozen in liquid nitrogen and stored at –80 C. Virus stocks were sequenced to confirm a lack of tissue culture adaptation in the polybasic cleavage site. Virus stocks and tissue homogenates were titered on Vero E6-TMPRSS2 cells.

### Mice and tamoxifen treatment.

C57BL/6 WT mice were obtained from The Jackson Laboratory. Casp11^–/–^ mice were provided by Junying Yuan (Harvard Medical School, Boston, Massachusetts). Cx3cr1-CreERT2 mice (JAX stock no. 021160) were obtained from The Jackson Laboratory. Casp11^fl/fl^ mice were provided by Timothy Millar (University of Pittsburgh, Pittsburgh, Pennsylvania).

For CX3CR1-MPS–specific CASP11 deletion experiments, Cx3cr1-CreERT2 mice were crossed with Casp11^fl/fl^ mice to generate Cx3cr1-CreERT2 Casp11^fl/fl^ experimental mice. Both control (Cx3cr1-CreERT2 Casp11^WT/WT^) and experimental (Cx3cr1-CreERT2 Casp11^fl/fl^) mice are homozygous for the Cx3cr1-CreERT2 knockin allele, rendering both groups CX3CR1-deficient. This shared genetic background ensures that phenotypic differences between groups are attributable specifically to CASP11 deletion rather than differential CX3CR1 expression. To rigorously control for potential off-target effects of Cre-recombinase expression and tamoxifen administration, Cx3cr1-CreERT2 Casp11^WT/WT^ mice were utilized as the primary control group, referred to as Cx3cr1-Cre controls throughout the manuscript. Both groups underwent an identical tamoxifen induction protocol prior to infection. Mice were administered tamoxifen at 75 mg/kg dissolved in corn oil via i.p. injection for 7 consecutive days, followed by a 3-day washout period prior to SARS-CoV-2 challenge. This abbreviated washout period was specifically selected to preserve the targeted circulating monocyte population, which possesses a relatively short physiological half-life, while allowing sufficient tamoxifen clearance.

Age-matched naive mice, housed under identical conditions without any experimental manipulation, were used as noninfected controls throughout all experiments. For BM chimera experiments, noninfected controls consisted of age-matched chimeric mice of the corresponding genotype that underwent identical irradiation and BM reconstitution but received no viral inoculation.

Experiments were conducted with approval from the IACUC at The Ohio State University, which is accredited by AAALAC International according to guidelines of the Public Health Service as issued in the *Guide for the Care and Use of Laboratory Animals* (National Academies Press, 2011).

### SARS-CoV-2 infection protocol.

Prior to infection, mice were anesthetized by isoflurane induction at 2%–3% concentration in an induction chamber for approximately 5–10 minutes until loss of righting reflex was confirmed. Mice were then infected i.n. with 50 μL of virus inoculum containing the indicated dose of MA10 SARS-CoV-2 delivered dropwise into both nares (25 μL per nare). Body surface temperature was measured daily using a Wahl HSI3000 Heat Spy Portable Thermal Imaging camera (Wahl Instruments) as a noninvasive measure of physiological status. Temperature values were normalized to each mouse’s preinfection baseline and expressed as percentage of original temperature. Mice were monitored daily for clinical signs of disease including body weight and temperature. Mice were weighed daily throughout the observation period. Humane endpoint criteria were defined as greater than 30% loss of initial body weight, labored breathing, or failure to ambulate.

### Mouse ages.

Global Casp11^–/–^ KO experiments (Figures 1–3) were performed using mice aged 3–4 months at the time of infection. BM chimera experiments (Figure 4) were performed using mice aged approximately 6 months at the time of infection, reflecting the additional time required for irradiation and full immune reconstitution. Cx3cr1-MPS-Casp11^–/–^ conditional KO experiments (Figure 5) were performed using mice aged 3–4 months at the time of infection.

### Plethysmography.

To accurately assess maximal respiratory distress while avoiding the confounding effects of survivor bias, comparative lung function analysis was strictly focused on the early acute phase (days 1–4 after infection). Because the highly susceptible WT mice began reaching terminal endpoints by day 5, analyzing physiological parameters beyond this point would artificially skew the WT cohort data by selectively reflecting only the most resilient survivors. Furthermore, days 3 and 4 correlate directly with the onset of peak clinical morbidity (maximal weight loss), making this the optimal and most representative window to evaluate the true physiological deficits induced by the viral infection prior to mortality.

### BM chimera mice.

Hematopoietic BM chimeric mice were generated using Casp11^–/–^ or WT BM cell donors and lethally irradiated WT hosts, generating Casp11^–/–^ → WT chimeras and WT → WT chimeras as described previously (81). Recipient mice were lethally irradiated with 2 doses of 6.5 Gy and reconstituted by tail vein injection of 2 × 10^6^ to 4 × 10^6^ donor BM cells. Recipients were allowed to reconstitute their circulating leukocyte pools for a minimum of 6 weeks prior to infection. At the time of infection, chimeric mice were approximately 6 months of age.

### GSEA.

GSEA was performed using the gseGO function in clusterProfiler (v4.10.1) on a ranked gene list ordered by log_2_ fold-change values derived from differential expression analysis, without pre-filtering of genes. Analyses included all 3 GO categories (Biological Process, Molecular Function, and Cellular Component) with the following parameters: nPerm = 10,000, minGSSize = 3, maxGSSize = 800, pvalueCutoff = 0.05, and Benjamini–Hochberg multiple testing correction (pAdjustMethod = “BH”). A normalized enrichment score (NES) indicates the direction and magnitude of enrichment. A positive NES indicates enrichment in the Casp11^–/–^, Casp11^–/–^ chimera, or Cx3cr1-MPS-Casp11^–/–^ experimental group relative to the respective control; a negative NES indicates enrichment in the control group. The top 20 enriched terms were visualized as dot plots, with dot color reflecting the Benjamini-Hochberg–adjusted *P* value and dot position on the *x* axis reflecting the number of genes contributing to the enrichment. The same GSEA pipeline was applied independently to RNA-Seq data from the global Casp11^–/–^ KO experiment, BM chimera experiment (Figure 4), and Cx3cr1-MPS-Casp11^–/–^ conditional KO experiment.

### Real-time qPCR.

Total RNA was extracted and reverse-transcribed to cDNA following the manufacturer’s protocol. qPCR was performed using SYBR Green chemistry with gene-specific primers for the SARS-CoV-2 nucleocapsid gene, *Il1b*, *Il6*, and *Cxcl1* (*KC*). Amplifications were carried out in 10–20 μL reactions on a real-time PCR system under standard cycling conditions. Gapdh was used as the reference gene. Relative copy number was calculated by normalizing target Ct values to *Gapdh* and then to control samples using the 2^–ΔΔCt^ method.

### Plaque assay.

The SARS-CoV-2 plaque assay was performed on Vero E6-TMPRSS2 cells in 12-well plates. Left lung lobes were homogenized in 1 mL sterile PBS and clarified by centrifugation. Plates were infected with 10-fold serial dilutions of lung homogenate supernatant. After absorption for 1 hour at 37°C, cells were overlaid with 1 mL of DMEM containing 0.25% (w/v) low-melting-temperature agarose, 0.12% (v/v) NaHCO_3_, 2% (v/v) FBS, 25 mM HEPES, 2 mM L-glutamine, 100 μg/mL streptomycin, and 100 U/mL penicillin. After incubation at 37°C for 24–48 hours, cells were fixed with 4% paraformaldehyde for 2 hours. The overlay was then removed and plaques were visualized by staining with 0.05% (v/v) crystal violet. Viral titers are expressed as PFUs per gram of lung tissue.

### Statistics.

Statistical analyses and data visualization were performed using GraphPad Prism software (v9.3.1). The specific statistical tests used are described in the individual figure legends and include Kaplan-Meier survival analysis with log-rank (Mantel-Cox) test, unpaired 2-tailed Student’s *t* test, 2-way ANOVA with Šídák’s multiple-comparison test, and 1-way ANOVA with Tukey’s multiple-comparison test. A *P* value less than 0.05 was considered statistically significant.

### Study approval.

All procedures were reviewed and approved by The Ohio State University BSL-3 Operations/Advisory Group, the Institutional Biosafety Officer, and the Institutional Biosafety Committee.

### Data availability.

RNA-Seq data have been deposited in NCBI’s Gene Expression Omnibus (GEO SuperSeries accession GSE329370), comprising SubSeries GSE329365 (global Casp11^–/–^ experiment, Figure 1), GSE329366 (Cx3cr1-MPS-Casp11^–/–^ experiment, Figure 5), and GSE329369 (BM chimera experiment, Figure 4 and Supplemental Figure 6). All RNA-Seq data are publicly available without restriction as of the date of publication. Values for all data points shown in graphs and values behind all reported means are provided in the Supporting Data Values file. Custom R scripts used for histology cellularity quantification and for RNA-Seq analysis are available from the corresponding author upon request. Any additional information required to reanalyze the data reported in this paper is available from the corresponding author upon request.

## Author contributions

MME and MMS designed and conducted experiments, analyzed data, validated reagents and mice, and wrote the manuscript. ODW, HMA, JRA, AB, JMH, GG, YYH, REM, RP, SEF, ADK, DB, JIO, SE, KPD, AY, MRJ, AMN, and MKC conducted experiments. MP, AW, and XZ analyzed data. MEP, EAH, HEG, SMN, ECB, JL, PNB, JSY, BMS, and PD provided critical reagents. AOA conceived the study, supervised the study, acquired funding, validated results, designed experiments, analyzed data, and wrote the manuscript.

## Conflict of interest

The authors have declared that no conflict of interest exists.

## Funding support

This work is the result of NIH funding, in whole or in part, and is subject to the NIH Public Access Policy. Through acceptance of this federal funding, the NIH has been given a right to make the work publicly available in PubMed Central.

- NIH grant R01AI159452 (to AOA).
- NIH grant R01HL168501 (to AOA).
- NIH grant P01AI175399 (to AOA).

## Supplementary Material

Supplemental data

Unedited blot and gel images

Supporting data values

## Version 1 09/22/2026

Electronic publication
