# Supplementary material for: Caspase-11–mediated hyperinflammation impairs CD8+ T cell immunity and viral clearance in severe SARS-CoV-2 infection
Source: JCI Insight. 2026 Sep 22;11(18):e199896. doi: 10.1172/jci.insight.199896 (PMC13596723; doi:10.1172/jci.insight.199896)
Supplement: Supplementary file 1 [file jciinsight-11-199896-s051.pdf]

A

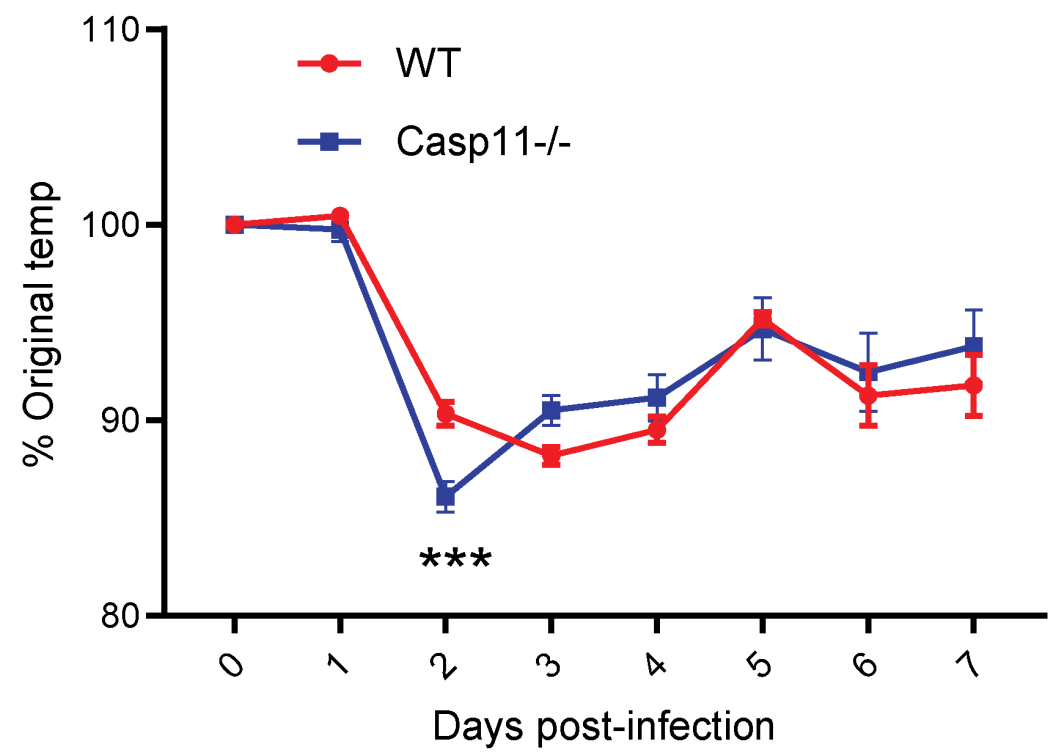

110
100
90
80
% Original temp
WT
Casp11-/-
***
0
1
2
3
4
5
6
7
Days post-infection

B

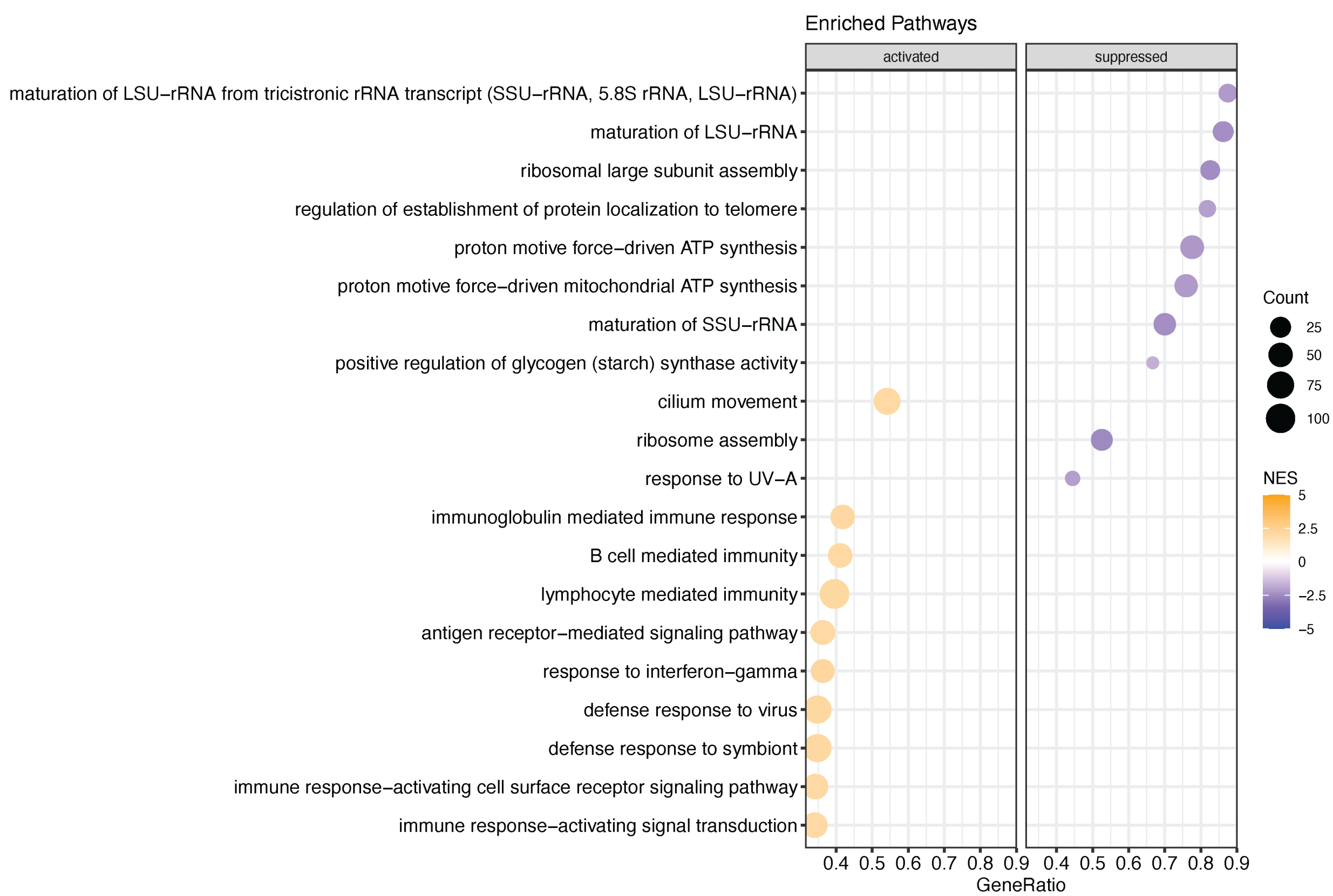

Enriched Pathways
activated
suppressed
maturation of LSU−rRNA from tricistronic rRNA transcript (SSU−rRNA, 5.8S rRNA, LSU−rRNA)
maturation of LSU−rRNA
ribosomal large subunit assembly
regulation of establishment of protein localization to telomere
proton motive force−driven ATP synthesis
proton motive force−driven mitochondrial ATP synthesis
maturation of SSU−rRNA
positive regulation of glycogen (starch) synthase activity
cilium movement
ribosome assembly
response to UV−A
immunoglobulin mediated immune response
B cell mediated immunity
lymphocyte mediated immunity
antigen receptor−mediated signaling pathway
response to interferon−gamma
defense response to virus
defense response to symbiont
immune response−activating cell surface receptor signaling pathway
immune response−activating signal transduction
0.4 0.5 0.6 0.7 0.8 0.9
0.4 0.5 0.6 0.7 0.8 0.9
GeneRatio
Count
25
50
75
100
NES
5
2.5
0
−2.5
−5

Supplementary Figure 1. CASP11 deficiency confers transient early temperature changes and enhances antiviral and lymphocyte-mediated immune responses during SARS-CoV-2 infection.

(A) Body temperature kinetics following severe SARS-CoV-2 infection. WT and Casp11^{-}/$^{-}$ mice were infected intranasally with 5×10^{5} TCID50 of MA10 SARS-CoV-2. Body surface temperature was monitored daily using a Wahl HSI3000 Heat Spy Portable Thermal Imaging Camera and expressed as a percentage of pre-infection baseline temperature. Casp11^{-}/$^{-}$ mice exhibited a transient but significant decrease in body temperature at day 2 post-infection compared to WT controls, with no significant differences observed at subsequent time points. Data are represented as mean ± SEM. Statistical significance was determined by two-way ANOVA with Šidák's multiple comparisons test. ***$p < 0.001$. (n = 20/WT, n = 18/Casp11^{-}/$^{-}$, equal proportions of male and female mice).

(B) Gene Set Enrichment Analysis (GSEA) of differential transcriptional responses. GSEA was performed on bulk RNA-seq data from lungs of Casp11^{-}/$^{-}$ and WT mice at day 4 post-infection. Enrichment plots show significant upregulation of pathways related to antiviral immunity, including the interferon-γ response, defense response to virus, and lymphocyte-mediated immunity in the Casp11^{-}/$^{-}$ group compared to the WT group. Additional enriched pathways include B cell and immunoglobulin-mediated responses, consistent with a shift toward adaptive immune activation in Casp11^{-}/$^{-}$ lungs. (n = 4/group, equal proportions of male and female mice).

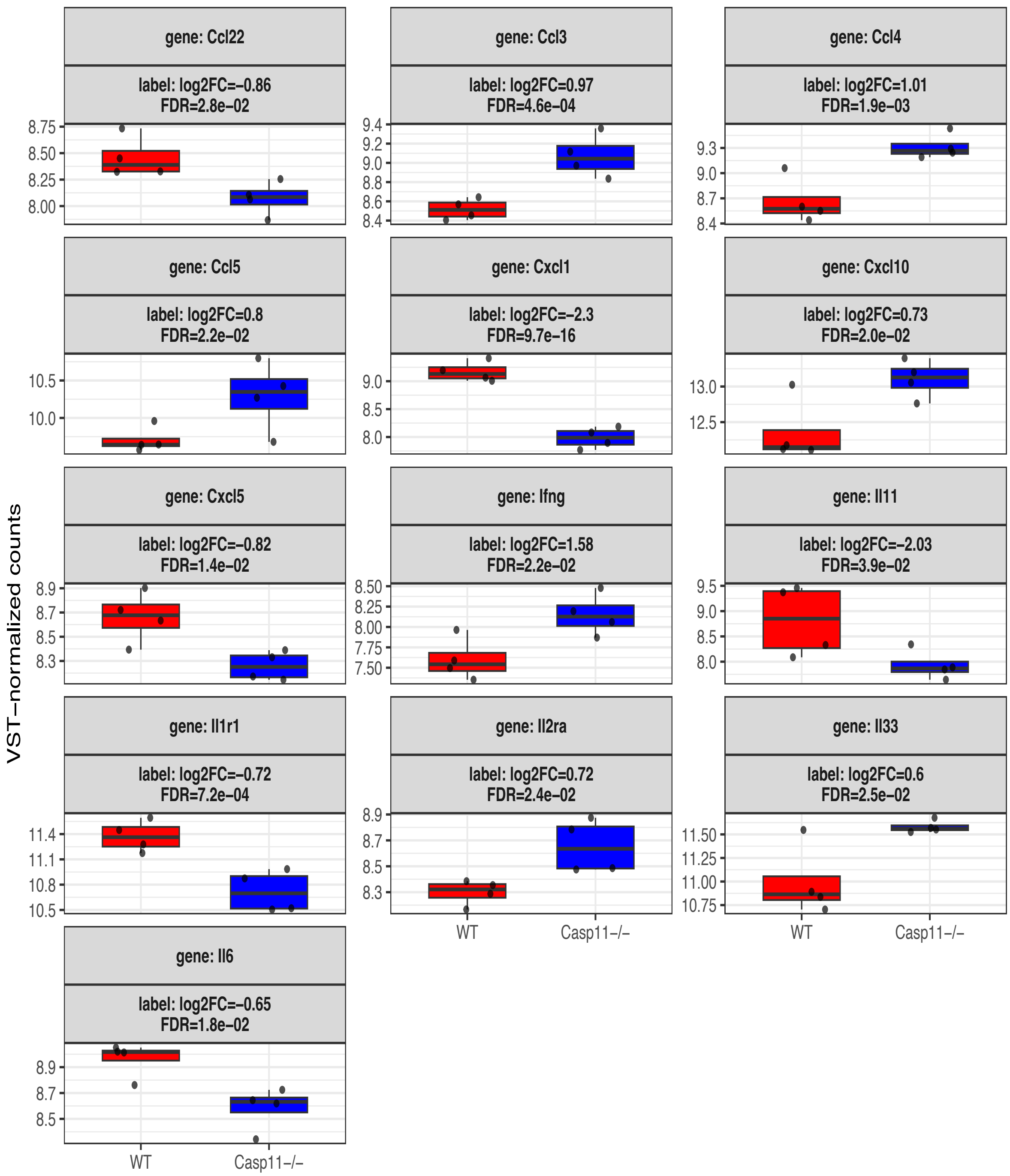

VST−normalized counts
gene: Ccl22
label: log2FC=−0.86
FDR=2.8e−02
gene: Ccl3
label: log2FC=0.97
FDR=4.6e−04
gene: Ccl4
label: log2FC=1.01
FDR=1.9e−03
gene: Ccl5
label: log2FC=0.8
FDR=2.2e−02
gene: Cxcl1
label: log2FC=−2.3
FDR=9.7e−16
gene: Cxcl10
label: log2FC=0.73
FDR=2.0e−02
gene: Cxcl5
label: log2FC=−0.82
FDR=1.4e−02
gene: Ifng
label: log2FC=1.58
FDR=2.2e−02
gene: Il11
label: log2FC=−2.03
FDR=3.9e−02
gene: Il1r1
label: log2FC=−0.72
FDR=7.2e−04
gene: Il2ra
label: log2FC=0.72
FDR=2.4e−02
gene: Il33
label: log2FC=0.6
FDR=2.5e−02
gene: Il6
label: log2FC=−0.65
FDR=1.8e−02
WT
Casp11−/−

Supplementary Figure 2. CASP11 deficiency reprograms cytokine and chemokine expression toward lymphocyte-promoting profiles during SARS-CoV-2 infection.

Expression profiles of significantly differentially expressed cytokine and chemokine genes in lungs of WT and Casp11$^{-/-}$ mice at day 4 post-infection. Plots show variance-stabilized transformed (VST) normalized counts and corresponding $\log_2$ fold change values, generated from DESeq2 differential expression (DE) analysis of bulk RNA-seq data using R. Casp11$^{-/-}$ lungs exhibited elevated expression of *Ifng*, *Ccl3*, *Ccl4*, *Ccl5*, and *Cxcl10*, associated with antiviral responses and effector T cell recruitment. In contrast, genes involved in neutrophil and monocyte recruitment and tissue inflammation, *Cxcl1*, *Cxcl5*, *Ccl22*, *Il6*, *Il11*, and *Il1r1*, were significantly downregulated. (n = 4/group, equal number of males and females per group).

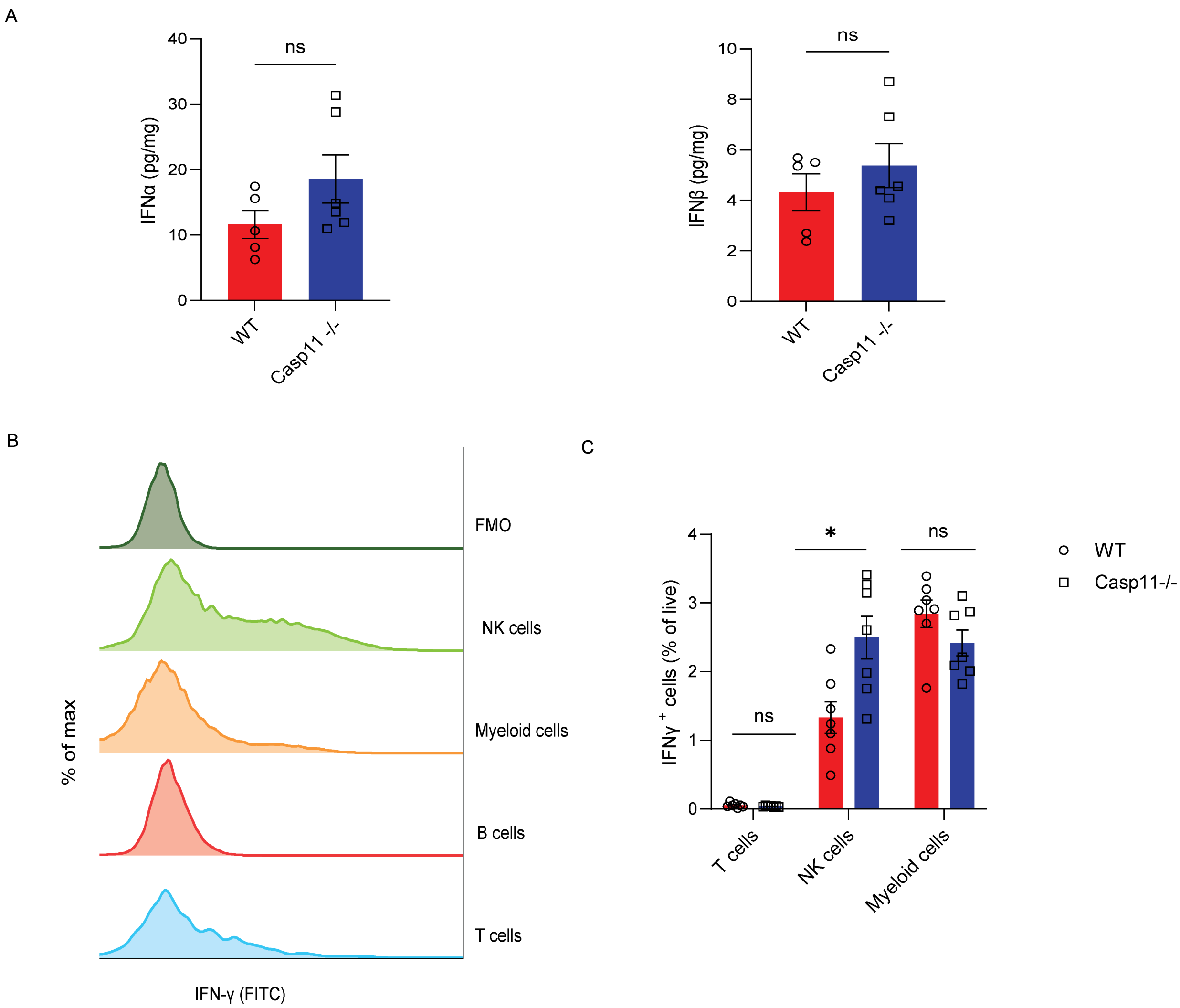

A
ns
IFNα (pg/mg)
0
10
20
30
40
WT
Casp11 -/-
ns
IFNβ (pg/mg)
0
2
4
6
8
10
WT
Casp11 -/-
B
FMO
NK cells
Myeloid cells
B cells
T cells
% of max
IFN-γ (FITC)
C
IFNγ + cells (% of live)
0
1
2
3
4
ns
*
ns
T cells
NK cells
Myeloid cells
WT
Casp11-/-

Supplementary Figure 3. CASP11 deficiency enhances NK cell-mediated IFN-γ production without altering type I interferon levels.

(A) Quantification of type I interferons, IFN-α (left) and IFN-β (right), in the lung homogenates of WT and Casp11^{-}/$^{-}$ mice at day 4 post-infection. Protein concentrations were measured by ELISA and expressed as pg/mg tissue. No statistically significant difference was observed between groups. (n = 5/WT, n = 6/Casp11^{-}/$^{-}$). Statistical significance was determined by unpaired Student's t-test; ns, not significant.

(B) Representative flow cytometric histograms evaluating intracellular IFN-γ production across distinct lung immune cell populations from WT lungs at day 4 post-infection, to identify the primary cellular source of early IFN-γ. Single-cell suspensions were stimulated ex vivo with PMA/ionomycin prior to intracellular cytokine staining. Fluorescence Minus One (FMO) control was utilized to establish the threshold for true IFN-γ positivity. Histogram data are presented as percentage of maximum peak height (% of max). NK cells exhibited a distinct rightward shift in IFN-γ fluorescence intensity compared to myeloid cells, B cells, and T cells.

(C) Quantification of IFN-γ^{+} cells (% of live cells) across T cell, NK cell, and myeloid cell populations in the lungs of WT (red bars, open circles) and Casp11^{-}/$^{-}$ (blue bars, open squares) mice at day 4 post-infection. Casp11^{-}/$^{-}$ mice demonstrate a statistically significant increase in IFN-γ-producing NK cells compared to WT controls, with no significant differences observed in T cell or myeloid cell populations. (n = 7/group, 4 males and 3 females). Data are represented as mean ± SEM. Statistical significance was determined by unpaired Student's t-test. *$p < 0.05$; ns, not significant.

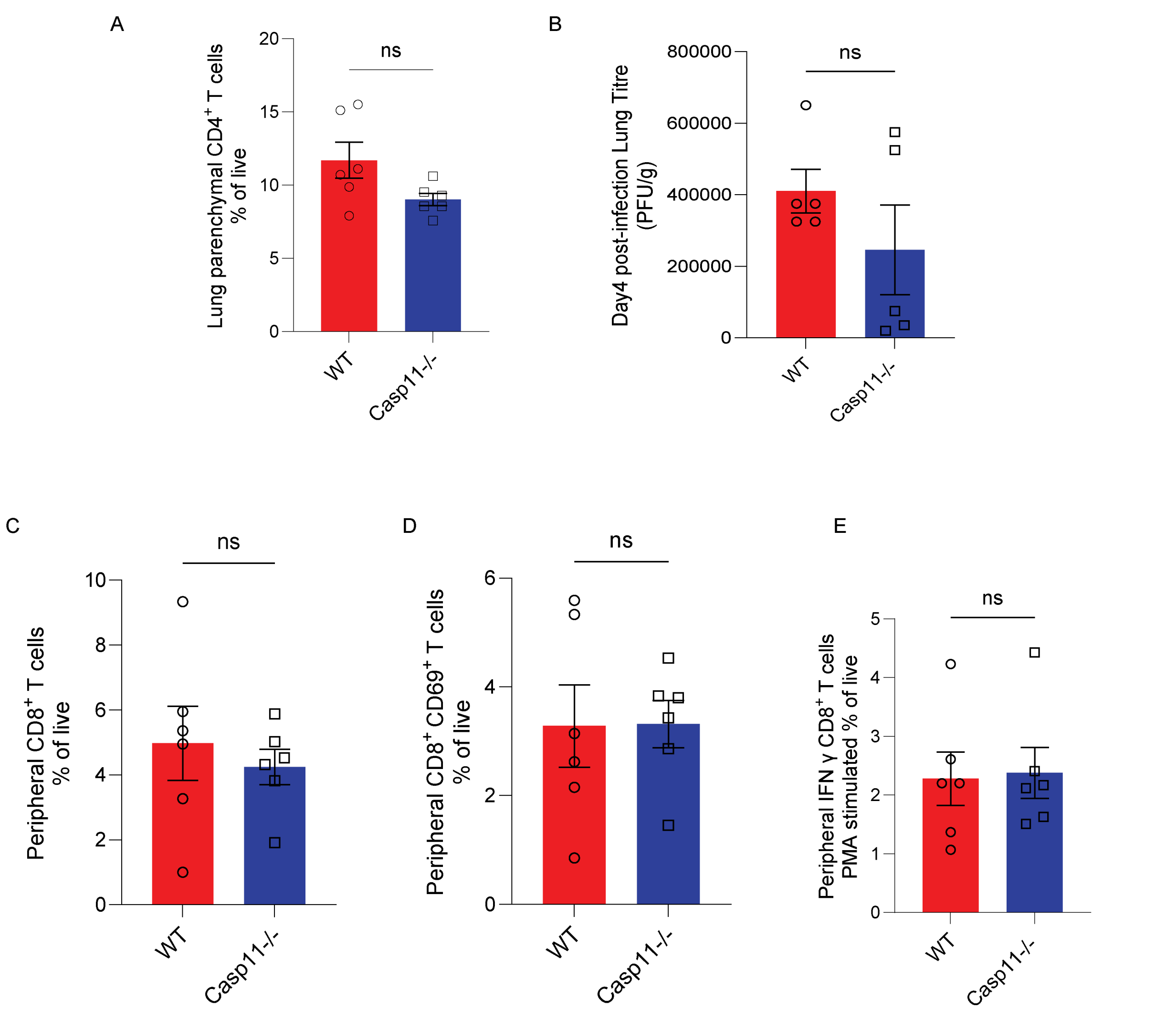

Supplementary Figure 4. Lung parenchymal CD4⁺ T cells, peripheral T cells, and early viral clearance are not altered by CASP11 deficiency.

(A) At day 7 post-infection, flow cytometry with intravascular anti-CD45 labeling revealed no significant difference in lung parenchymal ($CD45^-$) $CD4^+$ T cell numbers between

Casp11$^{-/-}$ and WT mice (n = 6/group, equal proportions of male and female mice). Unpaired t-test; ns, not significant.

(B) Viral titers in lung homogenates assessed by plaque assay at day 4 post-infection, expressed as PFU/g tissue. Both groups had comparable viral loads at day 4 (n = 5/group, 2 males and 3 females per group). Unpaired t-test; ns, not significant.

(C) Flow cytometric quantification of peripheral $CD8^+$ T cells ($CD45^+CD8^+$ as % of live cells) at day 7 post-infection.

(D) Flow cytometric quantification of peripheral activated $CD8^+$ T cells ($CD45^+CD8^+CD69^+$ as % of live cells) at day 7 post-infection.

(E) Flow cytometric quantification of peripheral IFN-γ^+ $CD8^+$ T cells ($CD45^+CD8^+$IFN-γ^+ as % of live cells) at day 7 post-infection following ex vivo PMA/ionomycin stimulation.

For panels (c-e), n = 6/group, equal proportions of male and female mice. Statistical significance was determined by unpaired t-test; ns, not significant.

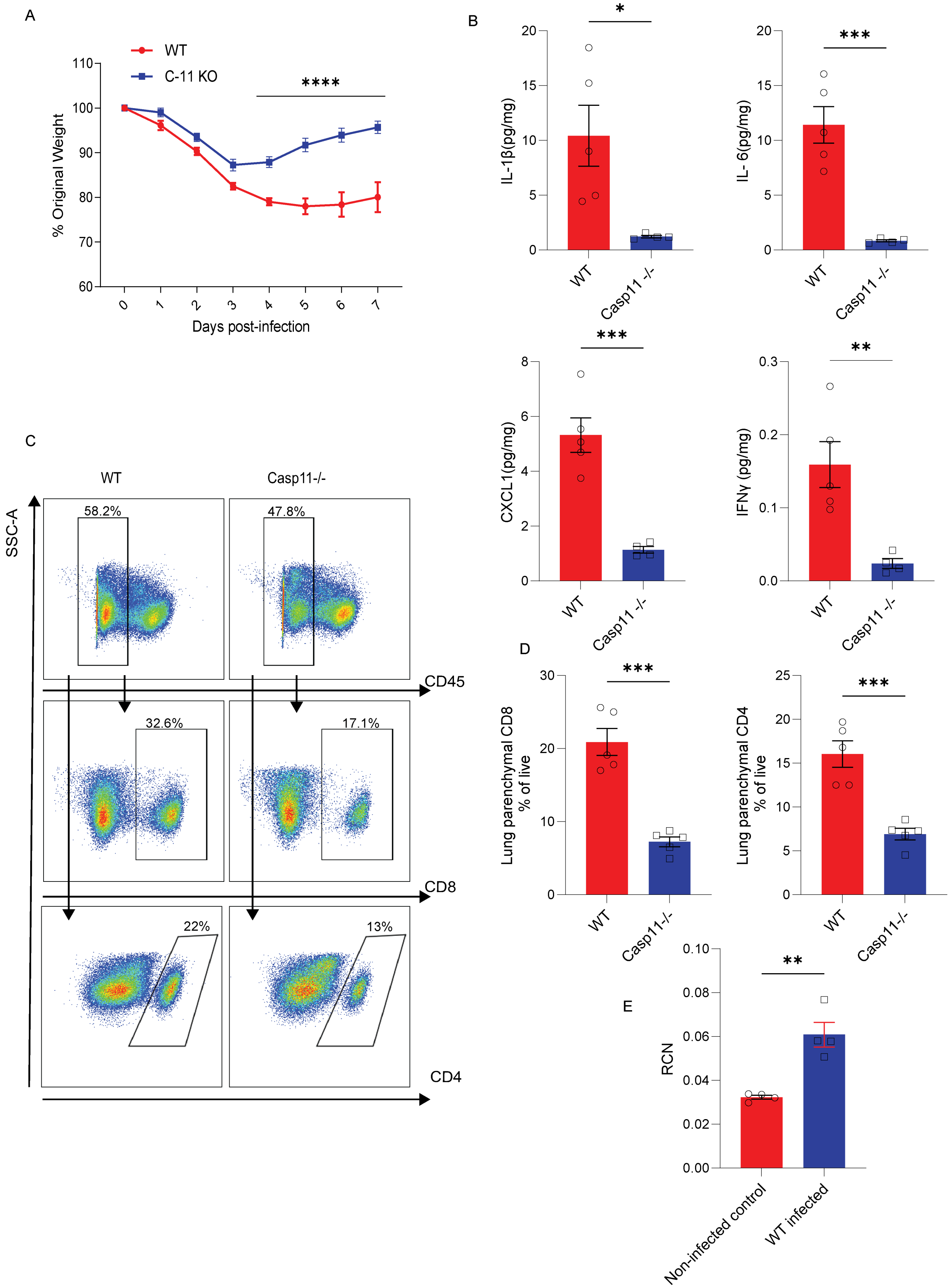
A
WT
C-11 KO
****
% Original Weight
Days post-infection
B
IL-1β(pg/mg)
IL- 6(pg/mg)
CXCL1(pg/mg)
IFNγ (pg/mg)
*
***
***
**
WT
Casp11 -/-
C
WT
Casp11-/-
SSC-A
58.2%
47.8%
CD45
32.6%
17.1%
CD8
22%
13%
CD4
D
Lung parenchymal CD8 % of live
Lung parenchymal CD4 % of live
***
***
WT
Casp11-/-
E
RCN
**
Non-infected control
WT infected

Supplementary Figure 5. CASP11 mediates persistent inflammatory and T cell responses after SARS-CoV-2 infection, indicating ongoing inflammation and immune activation despite viral clearance.

(A) Body weight change in WT and Casp11$^{-/-}$ mice following intranasal infection with 1×10^5 TCID50 of mouse-adapted (MA10) SARS-CoV-2. Both groups began regaining weight by day 4 post-infection, although WT mice exhibited a slower recovery trajectory compared to Casp11$^{-/-}$ mice. (n = 12 WT, n = 10 Casp11$^{-/-}$; equal proportions of male and female mice). Statistical analysis: two-way ANOVA with Šidák's multiple comparisons test. ****$p < 0.0001$.

(B) Cytokine protein levels measured by multiplex ELISA in lung homogenates at day 14 post-infection. WT mice exhibit significantly elevated IL-1β, IL-6, CXCL1, and IFN-γ compared to Casp11$^{-/-}$ mice, indicating persistent pulmonary inflammation in the presence of CASP11. Concentrations are expressed as pg/mg tissue. (n = 5 WT mice, 3 males and 2 females; n = 4 Casp11$^{-/-}$ mice, 2 males and 2 females). Statistical significance was determined by unpaired t-test.

(C-D) Flow cytometric analysis of lung parenchymal T cells at day 14 post-infection using intravascular CD45 labeling. Representative flow cytometry plots (C) show the sequential gating strategy: SSC-A vs CD45 to identify CD45^{-} parenchymal cells, followed by CD8 and CD4 T cell identification. Quantification (D) demonstrates significantly increased lung parenchymal CD8^{+} T cells and CD4^{+} T cells in WT compared to Casp11$^{-/-}$ mice, indicating sustained T cell activation in WT lungs despite viral clearance. (n = 5/group, 3 males and 2 females). Statistical significance was determined by unpaired t-test.

(E) *Casp11* mRNA expression in lung tissue quantified by RT-qPCR, showing significantly elevated Casp11 mRNA levels in WT infected mice compared to non-infected controls at 30 days post-infection. Results are expressed as relative copy number (RCN) normalized to Gapdh. (n = 4/group, equal proportions of male and female mice). Statistical significance was determined by unpaired t-test.

Data are represented as mean ± SEM. *$p < 0.05$; **$p < 0.01$; ***$p < 0.001$, ****$p < 0.0001$.

A

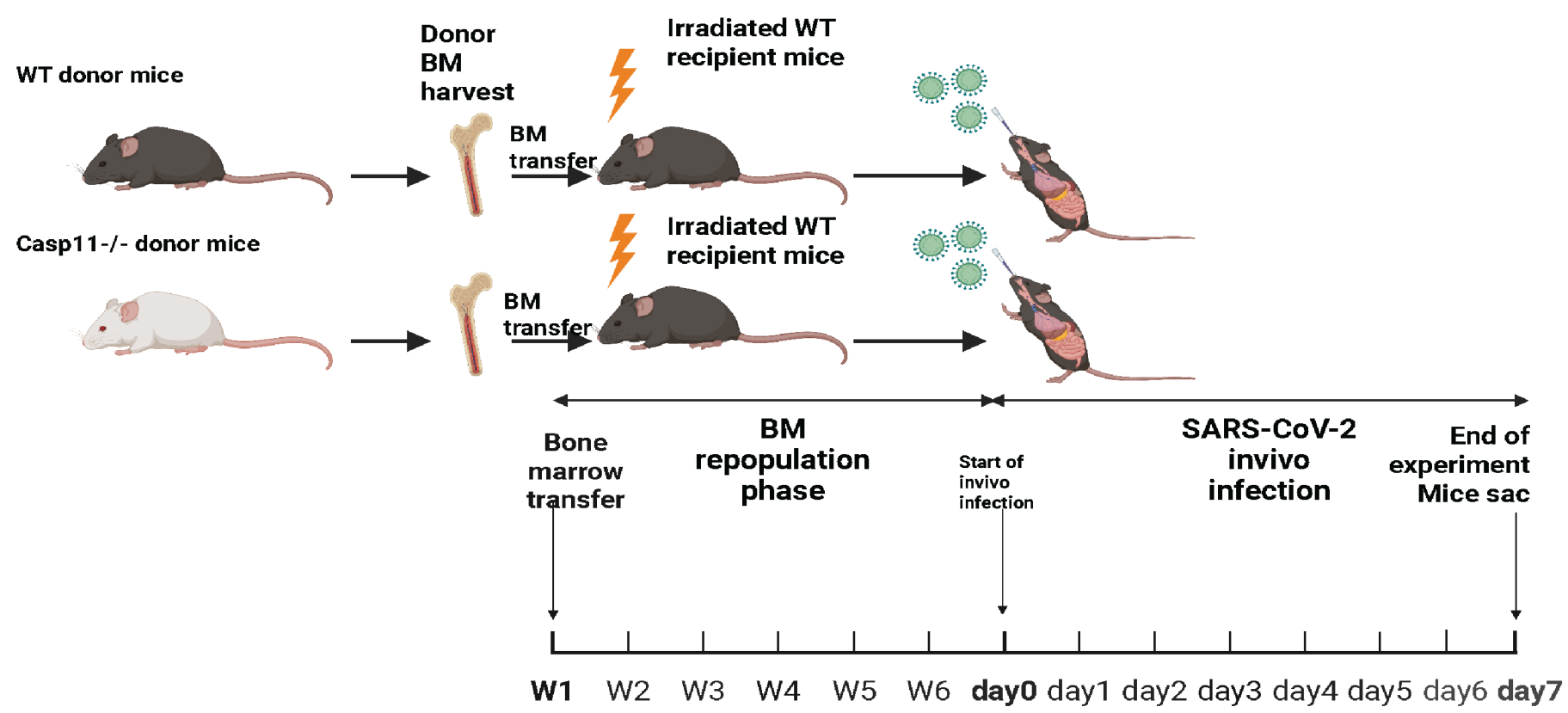

B

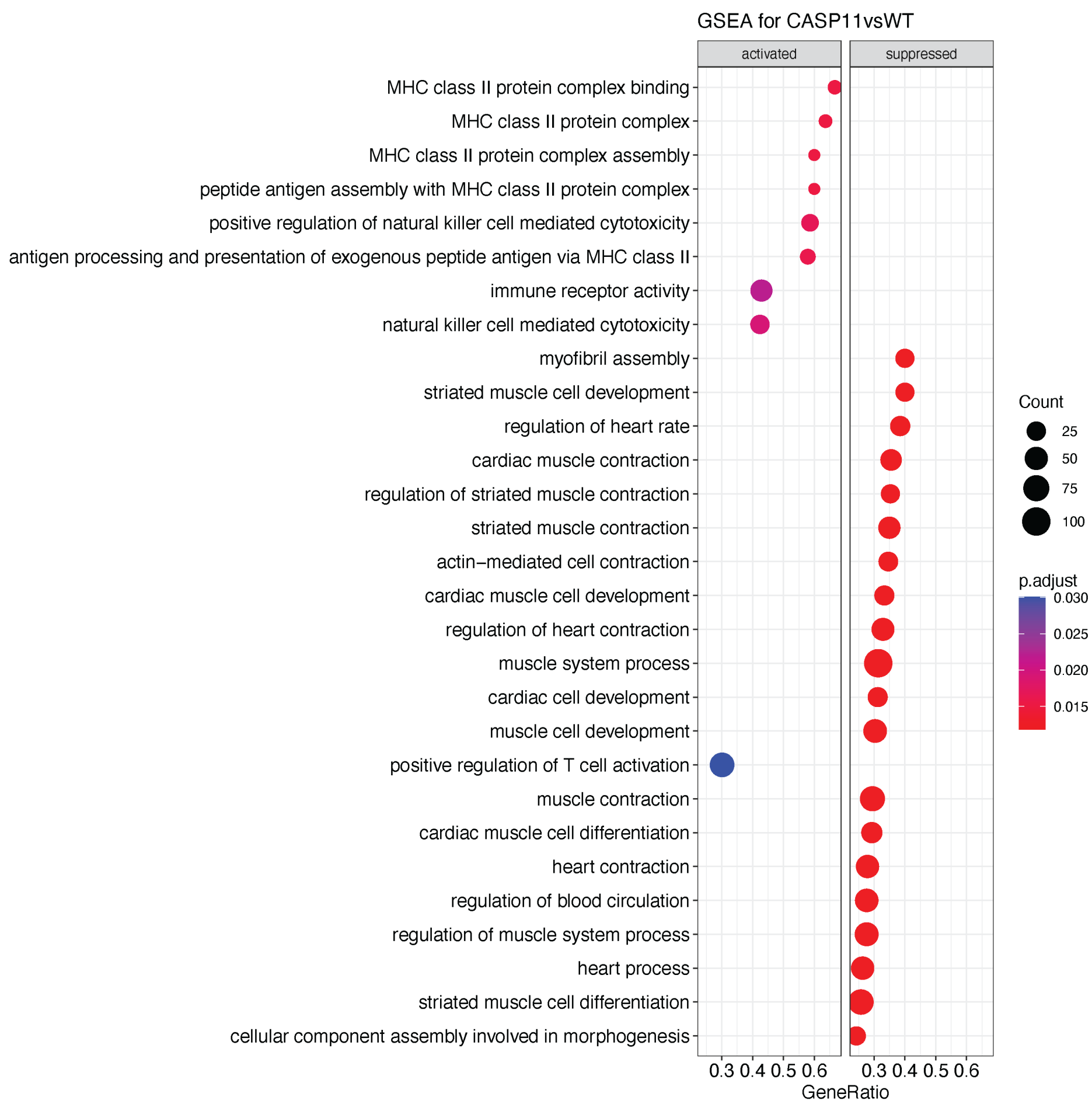

Supplementary Figure 6. CASP11 deficiency in hematopoietic cells enhances survival, reduces inflammation, and promotes T cell responses after SARS-CoV-2 infection.

(A) Schematic of bone marrow (BM) chimera generation. Lethally irradiated WT recipients were reconstituted with either WT or Casp11⁻/⁻ donor bone marrow, creating WT→WT

and $Casp11^{-/-}$→WT ($Casp11^{-/-}$ chimera) mice. This setup enabled assessment of hematopoietic CASP11 function while maintaining WT non-hematopoietic compartments, with alveolar macrophages remaining WT due to their radioresistant, self-renewing nature.

(B) Gene set enrichment analysis (GSEA) of RNA-seq data from lungs harvested at day 4 post-infection revealed upregulation of T cell activation and antigen presentation pathways in $Casp11^{-/-}$ chimeras, along with suppression of muscle contraction-related pathways. ($n = 4$/group, equal proportions of male and female mice). GSEA was performed using clusterProfiler with Benjamini-Hochberg multiple testing correction; pathways with adjusted $p < 0.05$ were considered significant.

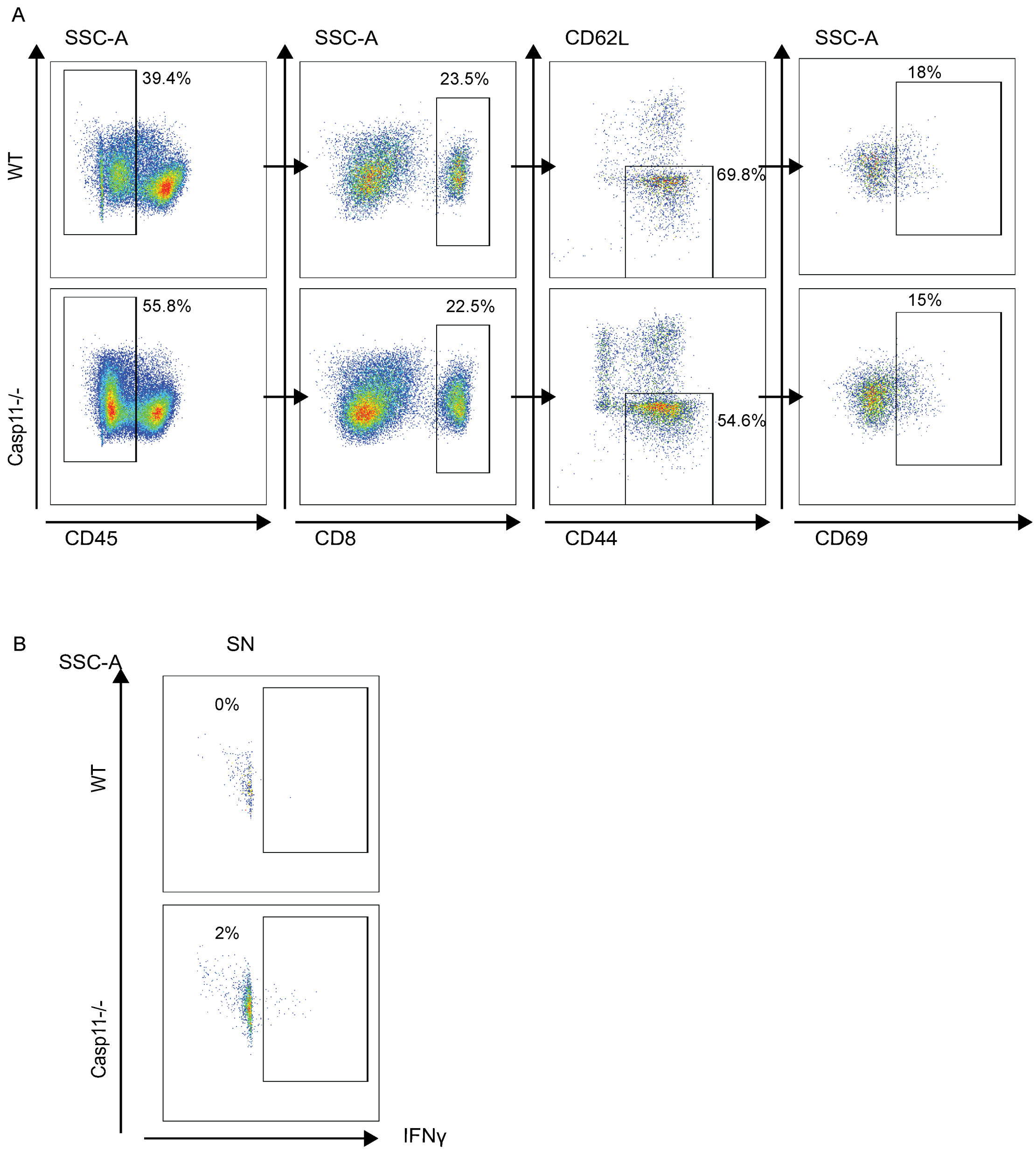
A
SSC-A
SSC-A
CD62L
SSC-A
39.4%
23.5%
69.8%
18%
WT
55.8%
22.5%
54.6%
15%
Casp11-/-
CD45
CD8
CD44
CD69
B
SSC-A
SN
0%
WT
2%
Casp11-/-
IFNγ

Supplementary Fig 7. Gating strategy of flow cytometry analysis of CD8 T cells in infected lungs of chimera mice

(A) Flow cytometric analysis of lung parenchymal $CD8^+$ T cells and activated effector $CD8^+$ T cells (CD62L^loCD44^hiCD69^hi) at day 7 post-infection.

(B) IFN-γ^+ $CD8^+$ T cells following ex vivo stimulation with spike+nucleocapsid (SN) peptide mix**.**

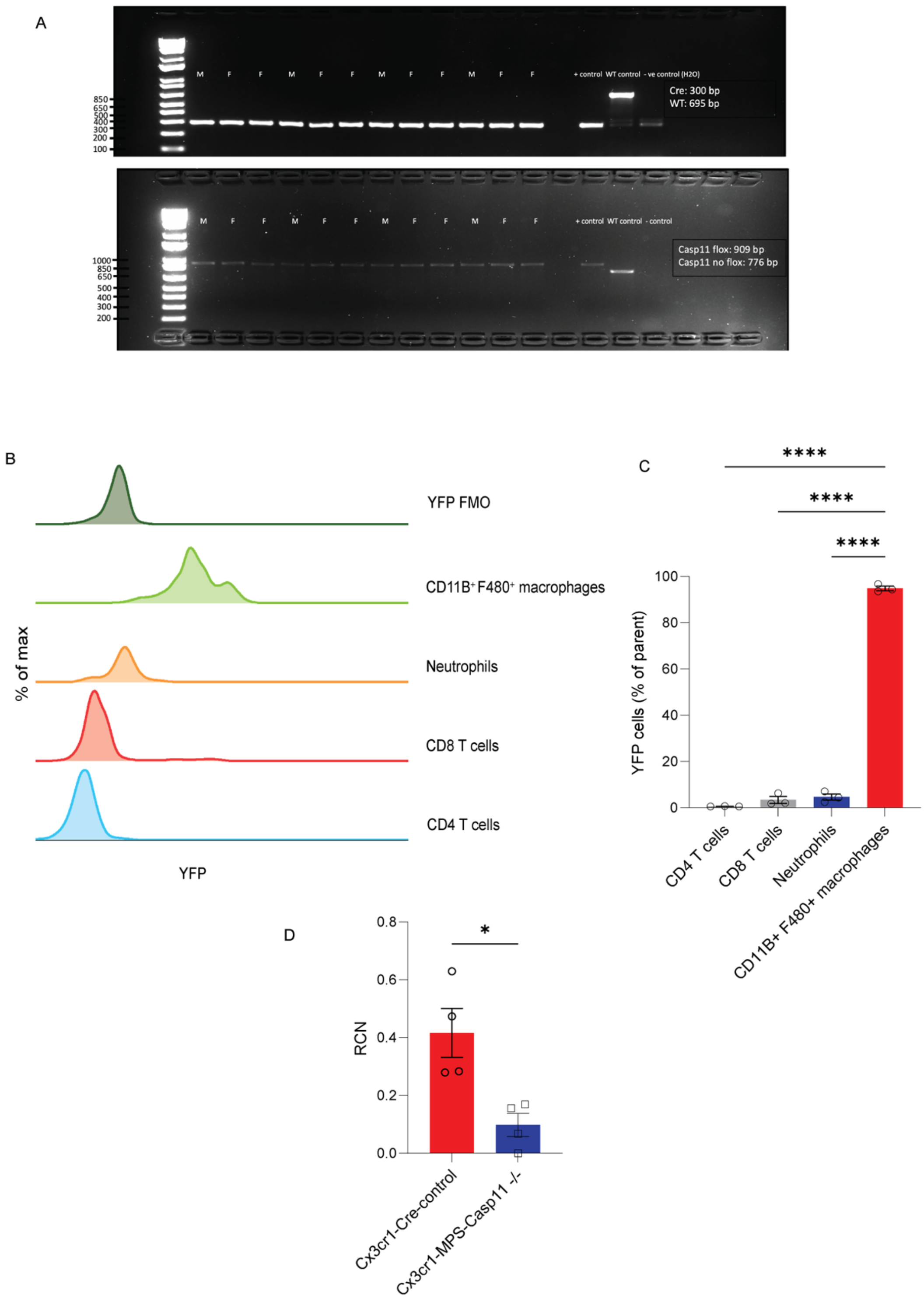
A
M F F M F F M F F M F F + control WT control - ve control (H2O)
Cre: 300 bp
WT: 695 bp
850
650
500
400
300
200
100
M F F M F F M F F M F F + control WT control - control
Casp11 flox: 909 bp
Casp11 no flox: 776 bp
1000
850
650
500
400
300
200
B
YFP FMO
CD11B⁺ F480⁺ macrophages
Neutrophils
CD8 T cells
CD4 T cells
% of max
YFP
C
****
****
****
YFP cells (% of parent)
100
80
60
40
20
0
CD4 T cells
CD8 T cells
Neutrophils
CD11B+ F480+ macrophages
D
*
RCN
0.8
0.6
0.4
0.2
0.0
Cx3cr1-Cre-control
Cx3cr1-MPS-Casp11 -/-

Supplementary Figure 8. Validation of specific Casp11 deletion within the lung mononuclear phagocyte system.

(A) Genotyping for the Cx3cr1-CreERT2 allele (Upper Panel). The mutant Cre knock-in allele yields a ~300 bp amplicon, while the wild-type Cx3cr1 allele yields a ~695 bp amplicon. Homozygous Cre-positive mice display only the 300 bp Cre band with absence of the WT band, whereas Cre-negative WT mice display only the 695 bp band. Genotyping for the Casp11 floxed allele (Lower Panel). The floxed allele is identified by a ~909 bp band, whereas the non-floxed WT allele yields a ~776 bp band. All experimental mice shown carry the homozygous Cre knock-in and homozygous floxed Casp11 alleles. Together, these panels confirm the successful generation and identification of Cx3cr1-CreERT2^{+}; Casp11^fl/fl (Cx3cr1-MPS-Casp11^{-}/$^{-}$) experimental mice and their Cre-matched controls used throughout the study.

(B) Representative flow cytometric histograms demonstrating YFP reporter expression across distinct lung immune cell subsets in Cx3cr1-MPS-Casp11^{-}/$^{-}$ non-infected lungs. Histograms show YFP fluorescence intensity (x-axis) as a percentage of maximum peak height (% of max) for each indicated population. The YFP FMO (fluorescence minus one) control establishes the threshold for true YFP positivity. CD11B^{+}F480^{+} macrophages show a strong rightward YFP^{+} shift compared to the FMO, confirming robust Cre-mediated recombination within the lung macrophage compartment. In contrast, neutrophils (LY6G^{+}), CD8^{+} T cells, and CD4^{+} T cells co-localize with the FMO control, confirming the absence of significant off-target recombination in these populations.

(C) Quantification of YFP reporter expression across pulmonary leukocyte subsets from Cx3cr1-MPS-Casp11^{-}/$^{-}$ mice. Data represent the mean percentage of YFP^{+} cells within each indicated parent population. CD11B^{+}F480^{+} macrophages, which include interstitial macrophages and monocyte-derived macrophages, demonstrate robust YFP positivity, confirming selective targeting of the Cx3cr1-expressing mononuclear phagocyte compartment. LY6G^{+} neutrophils, CD4^{+} T cells, and CD8^{+} T cells remain largely YFP^{-}, demonstrating a lack of significant off-target recombination. Data are represented as mean ± SEM. (n = 3/group). Statistical significance was determined by one-way ANOVA with Tukey's multiple comparisons test. ****$P < 0.0001$.

(D) Functional validation of Casp11 deletion during acute viral challenge. CD11B^{+}F480^{+} YFP^{+} lung macrophages were isolated via fluorescence-activated cell sorting (FACS) at day 3 post-SARS-CoV-2 infection. Quantitative PCR of Casp11 displayed as relative copy number (RCN) normalized to Gapdh (n = 4/group, 2 males and 2 females). Data are represented as mean ± SEM. Statistical significance was determined by unpaired Student's t-test. *$P < 0.05$.

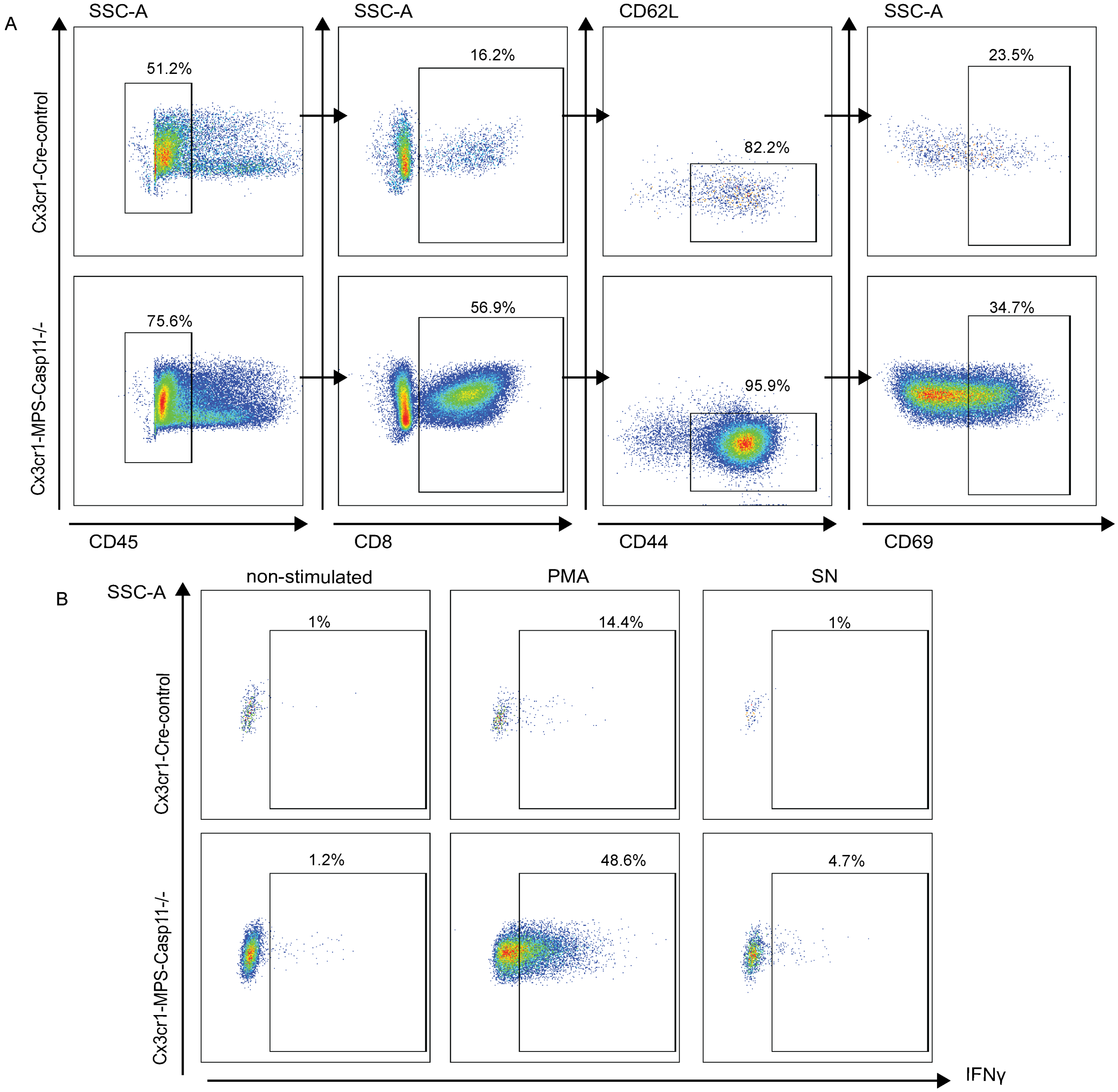

Supplementary Fig 9. Gating strategy of flow cytometry analysis of CD8$^+$ T cells in infected lungs of Cx3cr1-MPS-Casp11^{-}/$^{-}$ mice

(A) Flow cytometric analysis of lung parenchymal CD8$^+$ T cells and activated effector CD8$^+$ T cells (CD62L^loCD44^hiCD69^hi) at day 7 post-infection

(B) IFN-γ$^+$ CD8$^+$ T cells following ex vivo stimulation with PMA or spike+nucleocapsid (SN) peptide mix

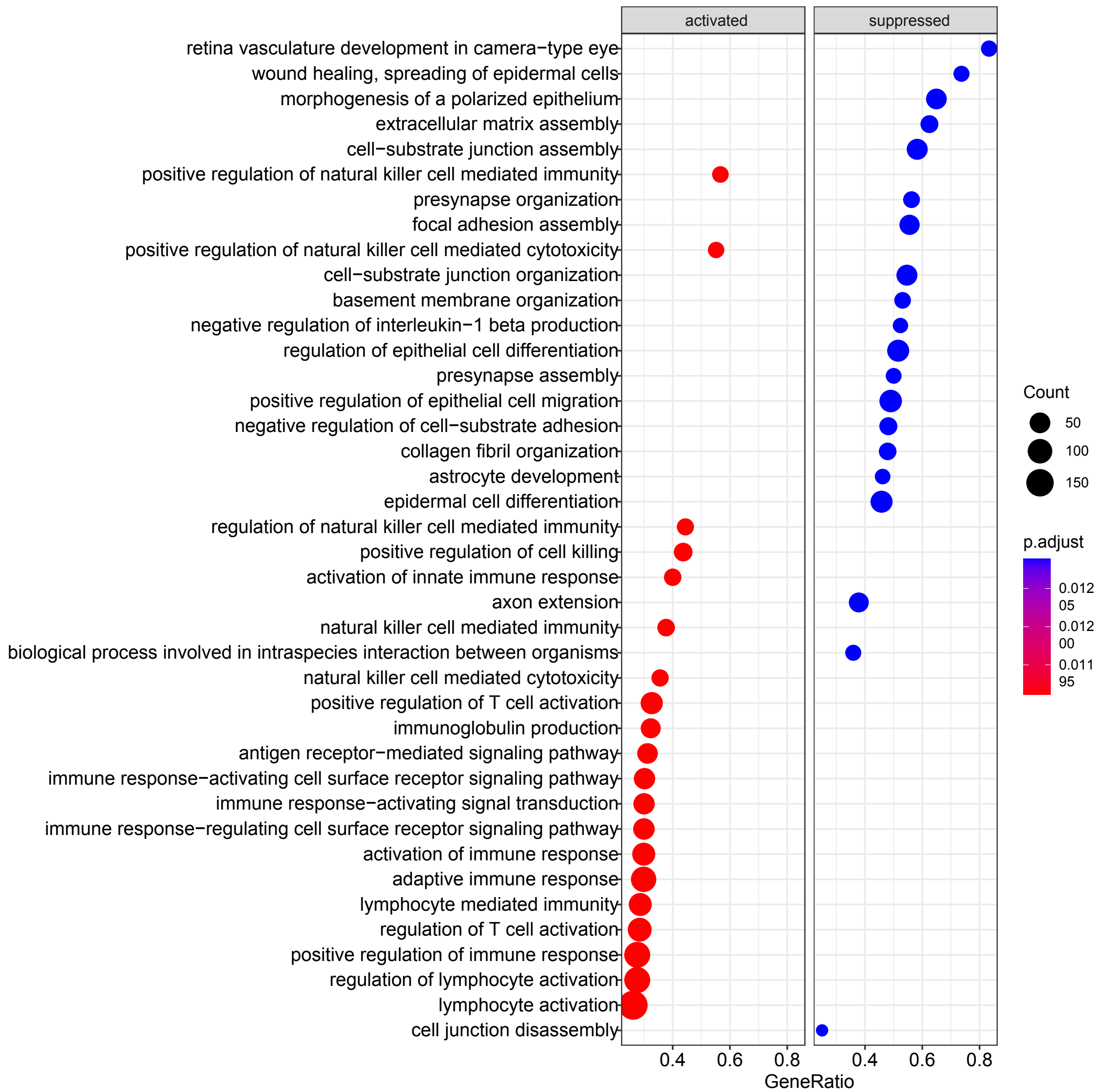

Supplementary Fig 10. Gene Set Enrichment Analysis (GSEA) of bulk RNA-seq data from lung tissue at day 4 post-infection in infected lungs of Cx3cr1-MPS-Casp11⁻/⁻ mice and Cx3cr1-Cre controls

Significant enrichment of antigen presentation, T cell activation, and adaptive immune response pathways in Cx3cr1-MPS-Casp11⁻/⁻ mice relative to Cx3cr1-Cre controls. (n = 5 males/group).

Materials and Methods (Continued)

Lung Harvest and Tissue Allocation

Following euthanasia, lungs were dissected into individual lobes and allocated to specific assays as follows. The right lung lobes (cranial, middle, caudal, and accessory) were used for flow cytometry and RT-qPCR analyses. The right middle lobe was specifically designated for histological analysis and processed for hematoxylin and eosin staining. The left lung lobe was used for ELISA, plaque assay, and RNA sequencing. For ELISA and plaque assays, the left lobe was weighed immediately after harvest and homogenized in 1 mL of sterile PBS. Homogenate supernatants were used for cytokine quantification and viral titer estimation and values were normalized to tissue weight, expressed as pg/mg or pg/g tissue.

CD45 Cell Labeling

To distinguish between lung parenchymal and intravascular T cell populations, anti-CD45-PE (clone 30-F11, BD Biosciences; 3 μg in 100 μL sterile PBS) was retro-orbitally injected 10 minutes prior to euthanasia to label circulating lymphocytes, while resident lung parenchymal lymphocytes remained unlabeled (7, 8). Peripheral blood collected at the time of sacrifice confirmed >90% labeling of circulating lymphocytes by flow cytometry.

Tissue Dissociation and Flow Cytometry

Right lung lobes were dissociated into single-cell suspensions using the gentleMACS Octo Dissociator and Miltenyi Lung Dissociation Kit (Miltenyi Biotec, Cat. 130-095-927). Red blood cells were lysed by incubating cells in 2 mL ACK lysis buffer for 5 minutes at room temperature. After lysis, cells were washed in DPBS containing 1% BSA. The single-cell suspension was centrifuged and the cell pellets were washed twice with PBS. Cells were resuspended in T cell medium (RPMI 1640 supplemented with 0.1% gentamicin, 10% heat-inactivated FBS, GlutaMAX, and 5×10^{-5} M β-mercaptoethanol) and incubated for 4–5 hours at 37°C in the presence of a protein transport inhibitor cocktail (eBioscience). Stimulation was performed either nonspecifically with PMA (50 ng/mL) and

ionomycin (500 ng/mL), or with SARS-CoV-2 peptide pools (Miltenyi Biotec) at 1 μg/mL per peptide. These included PepTivator® SARS-CoV-2 Prot_S+, PepTivator® SARS-CoV-2 Prot_S (Refs. 130-127-312, 130-126-700), and when indicated, PepTivator® Prot_N (Ref. 130-126-698).

Cells incubated with DMSO alone served as negative controls. For some experiments, a combination of Spike and Nucleocapsid peptides was used for stimulation.

Following stimulation, cells were washed with cold PBS and stained with Live/Dead Zombie NIR Fixable Viability Dye (BioLegend, Cat. 423105) or LIVE/DEAD™ Fixable Aqua Dead Cell Stain Kit (Invitrogen, Cat. L34957) for 30 minutes at 4°C. After two washes in FACS buffer (PBS with 1% FBS), cells were resuspended in Fc Block (clone 93; eBioscience, Cat. 14-0161-86) for 5 minutes at 4°C, then stained with the following surface antibodies for 20 minutes at 4°C: CD3 V450 (clone 17A2; BD Biosciences, Cat. 561389), CD8 AF700 (clone 53-6.7; BD BioLegend, Cat. 100730), CD4 BV750 (clone H129.19; BD Biosciences, Cat. 747275), CD44 PerCP-Cy5.5 (clone IM7; BD Biosciences, Cat. 560570), CD62L BV605 (clone MEL-14; BD Biosciences, Cat. 563252), and CD69 BV711 (clone HI.2F3; BD Biosciences, Cat. 740664). Cells were then fixed with intracellular fixation buffer (eBioscience, Cat. 00-8222-49) for 20 minutes at room temperature, permeabilized (eBioscience, Cat. 00-8333-56), and stained intracellularly for 30 minutes at 4°C with IFN-γ FITC (clone XMG1.2; eBioscience, Cat. 11-7311-82). Fluorescence minus one and isotype controls were included as negative controls. For tetramer staining experiments, H-2Db N219-227 tetramer (NIH Tetramer Core Facility) was used.

ELISAs

Multiplex cytokine analysis of IL-1β, IL-6, CXCL1, and IFN-γ was performed using the Meso Scale Discovery (MSD) platform (Cat. K15255D). Type I interferons (IFN-α and IFN-β) were quantified by conventional sandwich ELISA using commercially available kits (Invitrogen, Cat. 50246672, for IFN-α; R&D Systems, Cat. DY823405, for IFN-β) according to the manufacturers' instructions. The left lung lobe was weighed immediately after harvest, homogenized in 1 mL sterile PBS, and centrifuged to obtain clarified supernatant. Cytokine concentrations were normalized to lung tissue weight and expressed as pg/mg tissue. For samples generated in the BSL-3 facility, lung homogenate supernatants were decontaminated by UV irradiation in accordance with OSU BSL-3 biosafety guidelines prior to analysis.

Histology and Image Analysis

The right middle lung lobe was removed from infected mice and fixed in 10% formalin at room temperature. Sample preparation, processing, and hematoxylin and eosin (H&E) staining were performed by Histowiz, Inc. (Brooklyn, NY). H&E images were analyzed for tissue cellularity using a custom R script developed in-house. Briefly, images were converted to grayscale and global thresholding was applied to identify white spaces. Connected components corresponding to large empty regions were excluded based on a predefined size threshold. Morphological operations (convex hull filling, closing, and dilation) were applied to refine the tissue mask. Adaptive thresholding was then performed within the masked region to identify cellular structures. Cellularity was quantified as the ratio of cellular area to total tissue area, expressed as a percentage.

RNA Extraction, Purification, and Sequencing

Total RNA was extracted from the left lung lobe using TRIzol reagent (Thermo Fisher Scientific, Cat. 15596026) according to the manufacturer's instructions. RNA cleaning and concentration were performed using the Zymo Research RNA Clean & Concentrator-5 kit (Cat. R1015) following the manufacturer's protocol. For RNA extraction from FACS-sorted cell populations, total RNA was extracted using the PureLink RNA Mini Kit (Thermo Fisher Scientific) according to the manufacturer's instructions. RNA quantity was assessed using a Qubit 4.0 Fluorometer (Thermo Fisher Scientific) and RNA integrity was assessed using TapeStation (Agilent Technologies).

Library preparation and sequencing were performed at GENEWIZ from Azenta Life Sciences (South Plainfield, NJ, USA). cDNA libraries were generated using the NEBNext Ultra II RNA Library Prep Kit for Illumina with the NEBNext Poly(A) mRNA Magnetic Isolation Module (New England Biolabs, Ipswich, MA, USA) according to the manufacturer's recommendations. Briefly, mRNAs were enriched using Oligo-d(T) beads, fragmented for 15 minutes at 94°C, and used as template for first- and second-strand cDNA synthesis. cDNA fragments were end-repaired and adenylated at 3' ends, followed by ligation of universal adapters, index addition, and library enrichment by limited-cycle PCR. Library quality and quantity were assessed using the Agilent TapeStation (Agilent Technologies), Qubit 4.0 Fluorometer (Thermo Fisher Scientific), and quantitative PCR (KAPA Biosystems). Sequencing libraries were multiplexed and clustered onto a flowcell on the Illumina NovaSeq instrument and sequenced using a 2×150 bp paired-end (PE) configuration. Image analysis and base calling were conducted by the NovaSeq Control Software (NCS). Raw sequence data (.bcl files) were converted into FASTQ files and

demultiplexed using Illumina bcl2fastq 2.20 software, allowing one mismatch for index sequence identification.
