# Supplementary material for: Caspase-11–mediated hyperinflammation impairs CD8+ T cell immunity and viral clearance in severe SARS-CoV-2 infection
Source: JCI Insight. 2026 Sep 22;11(18):e199896. doi: 10.1172/jci.insight.199896 (PMC13596723; doi:10.1172/jci.insight.199896)
Supplement: Supplementary file 2 [file jciinsight-11-199896-s052.pdf]

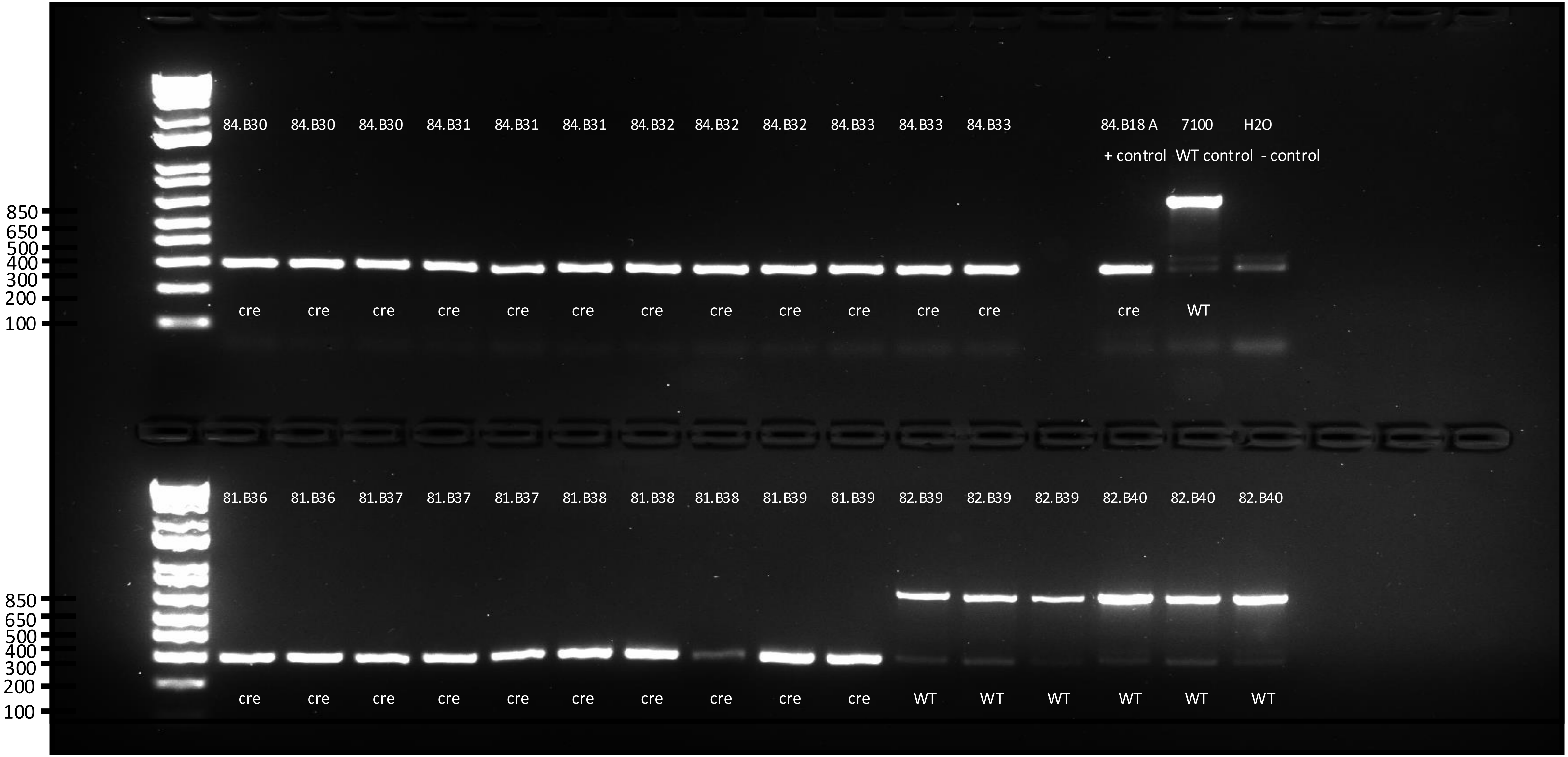

Cre: 300 bp
WT: 695 bp

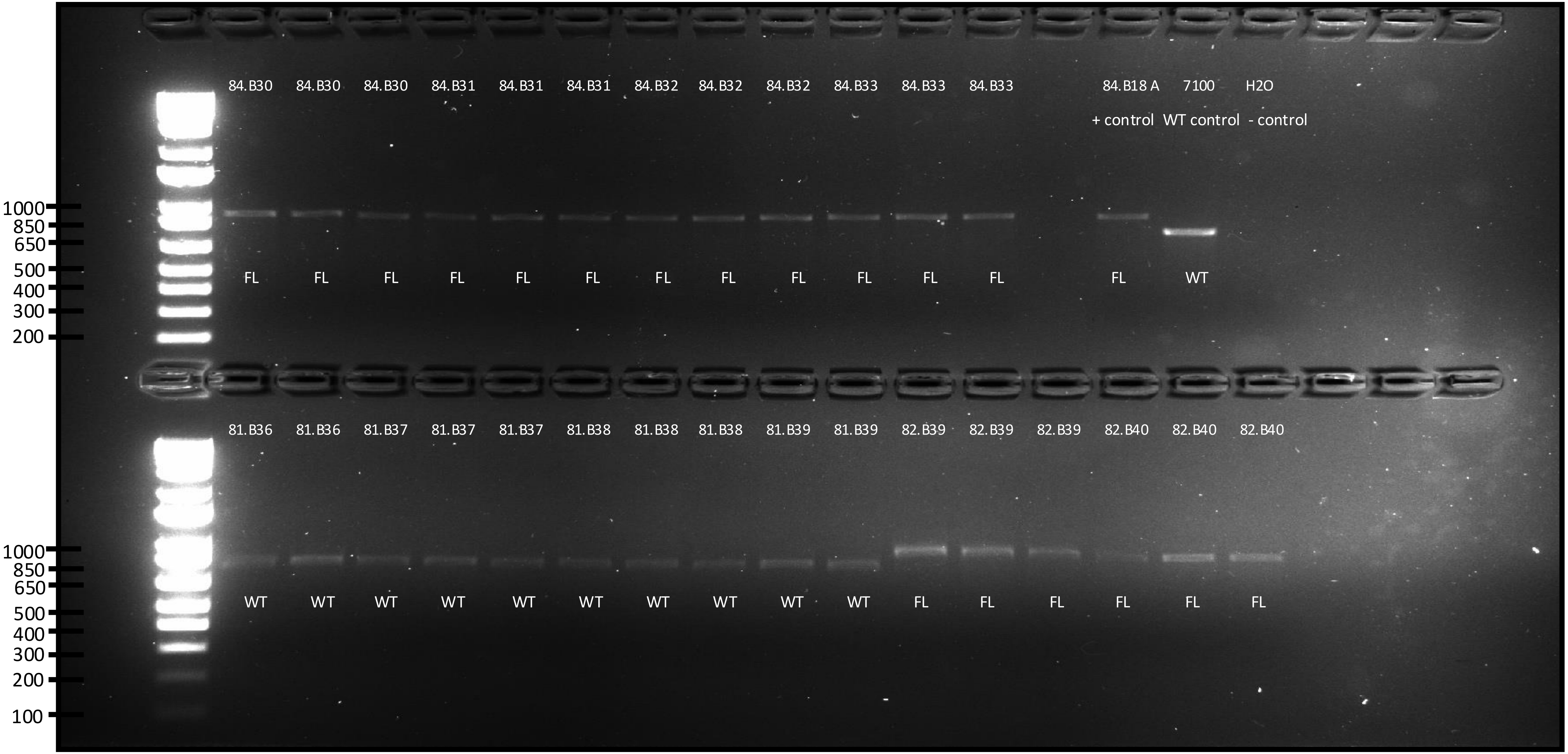

C-11 Flox: 909 bp
WT: 776 bp
